# Volatile Molecule Profiles and Anti-*Listeria monocytogenes* Activity of Nisin Producers *Lactococcus lactis* Strains in Vegetable Drinks

**DOI:** 10.3389/fmicb.2019.00563

**Published:** 2019-03-26

**Authors:** Lorenzo Siroli, Lucia Camprini, Maria Barbara Pisano, Francesca Patrignani, Rosalba Lanciotti

**Affiliations:** ^1^Department of Agricultural and Food Sciences, Alma Mater Studiorum, University of Bologna, Cesena, Italy; ^2^Interdepartmental Center for Industrial Agri-food Research, University of Bologna, Cesena, Italy; ^3^Department of Medical Sciences and Public Health, University of Cagliari, Monserrato, Italy

**Keywords:** *Lactococcus lactis*, nisin, antimicrobial activity, vegetable drinks, soymilk, carrot juice

## Abstract

This work aimed to evaluate the potential of 15 nisin producing *Lactococcus lactis* strains, isolated from dairy products, for the fermentation of soymilk and carrot juice. In particular, the acidification and the production of nisin in the food matrices were recorded. Moreover, three strains (LBG2, FBG1P, and 3LC39), that showed the most promising results were further scrutinized for their anti-*Listeria monocytogenes* activity and volatile molecules profile during fermentation of soymilk and carrot juice. *Lactococcus lactis* strains LBG2, FBG1P, and 3LC39 resulted the most interesting ones, showing rapid growth and acidification on both food matrices. The higher amounts of nisin were detected in soymilk samples fermented by the strain LBG2 after 24 and 48 h (26.4 mg/L). Furthermore, the rapid acidification combined with the production of nisin resulted in a strong anti-*Listeria* activity, reducing the pathogen loads below the detection limit, in carrot juice samples fermented by the strains LBG2 and FBG1P and in soymilk by the strain LBG2. The fermentation increased the presence of volatile molecules such as aldehydes and ketones with a positive impact on the organoleptic profile of both the fermented products. These results highlighted the interesting potential of three nisin producing *L. lactis* strains for the production of fermented carrot juice and soymilk. In fact, the fermentation by lactic acid bacteria, combined or not with other mild technologies, represents a good strategy for the microbiological stabilization of these products. Furthermore, the increase of molecules with a positive sensory impact, such as aldehydes and ketones, in the fermented products suggests a possible improvement of their organoleptic characteristics.

## Introduction

Vegetable beverages are perceived by consumers as “healthy” foods because of their low content of sodium, cholesterol, fat and because they are rich in vitamin C, polyphenols and flavonoids that contribute to the good antioxidant properties of these products (Kumar et al., 2009; Patrignani et al., 2009). For these reasons, their market is increased in recent years (Kregiel, 2015). Among vegetable drinks, soymilk, and soy products are certainly the most requested and widespread among western consumers (Terhaag et al., 2013; Riciputi et al., 2016). Soymilk is an easily digestible food and it is generally characterized by high protein content, a moderate ratio of β-conglycinin (7 S) and glycinin (11 S) and high sugar content (Kaneko et al., 2011). Protein and fatty components in soybeans are high in containing all the essential amino acids in amounts that closely match those required by humans. Also, soybean lipids contain a high proportion of unsaturated fatty acids, including oleic, linoleic, linolenic acids, and minor lipid components as phospholipids, phytosterols, and tocopherols with recognized health-promoting features (Serrazanetti et al., 2013; Medic et al., 2014). In addition, soy-based foods have received increased attention in health-conscious societies, especially in Western countries, due to their reported beneficial effects on menopausal symptoms and metabolic-related diseases (Cao et al., 2018).

Among vegetable-based beverages, carrot juice has increased consumption in various countries in recent years (Sharma et al., 2012). Carrot juice is one of the most popular non-alcoholic beverages consumed in Germany and northern European countries (Sharma et al., 2012). It is an important natural source of antioxidants such as α and β-carotene, precursors of vitamin A and polyacetylene which have beneficial health and anti-tumor activities (Pferschy-Wenzig et al., 2009; Sharma et al., 2012; Zhang et al., 2016). Also, its antianemic activity and benefits in the healing of wounds are documented (Nadeem et al., 2018). Carrot and soy beverages due to their high pH and the high content in sugars can favor the growth of spoilage and pathogenic microorganisms (Patrignani et al., 2009). Recent outbreaks of food-borne diseases have also been attributed to unpasteurized juices having lower pH-values such as orange and apple juices contaminated with pathogenic agents such as *Salmonella* spp., *Escherichia coli* O157: H7 and *Listeria monocytogenes* demonstrating that unpasteurized juices can be a vehicle for foodborne outbreaks (Berger et al., 2010; Callejón et al., 2015). In recent years, the need to increase shelf-life of such products, without detrimental effect on sensory, functional and nutritional properties, has generated, a growing interest in the application of mild heat treatments in combination with the refrigeration or non-thermal technologies including UV treatments, pulsed electric fields high hydrostatic pressures (Bello et al., 2014) and high pressures homogenization (HPH) (Kubo et al., 2013; Patrignani et al., 2013) and lactic fermentation of vegetable matrices (Karovičová and Kohajdová, 2005; Tamminen et al., 2013; Mauro et al., 2016; Timmermans et al., 2016; Tremarin et al., 2017; Bevilacqua et al., 2018).

Among these alternative methods, lactic acid fermentation, applied as a preservation method for the production of finished and semi-finished products, is considered an important technology due to the increasing quantity of raw materials processed in this way in the food industry (Di Cagno et al., 2013). The main reasons for this interest are the nutritional, physiological and hygienic aspects of the process and the related costs of implementation and production (Karovičová and Kohajdová, 2005). Literature data report the increase in fermented vegetable juices produced mainly from cabbage, red beets, carrots, celery and tomatoes (Di Cagno et al., 2013; Filannino et al., 2014).

For centuries, fermented soy foods have been dietary staples in Asia and, now, in response to consumer demand, they are available throughout the world (Cao et al., 2018). On the other hand, fermentation of soy bestows unique flavors, boosts nutritional values and increases or adds new important functional properties. It is reported that fermentation has been used to increase the bioavailability of vitamins, minerals and isoflavones in soy, as well as to modify its flavor, improve its stability and even create new food products (Rekha and Vijayalakshmi, 2010; Riciputi et al., 2016). The fermentation is widely recognized as a pivotal tool to increase the safety, shelf-life and functional and sensory properties of many other vegetables based beverages particularly when managed with tailored starter cultures (Serrazanetti et al., 2013; Mauro et al., 2016; Riciputi et al., 2016). Currently, the fermentation processes of vegetable-based juices and beverages in Europe, are guided using well-characterized commercial microbial strains belonging to species *Lactobacillus plantarum, Lactobacillus bavaricus, Lactobacillus xylosus, Lactobacillus brevis, Lactobacillus reuteri, Bifidobacterium lactis, Bifidobacterium bifidum* (Karovičová and Kohajdová, 2005; Mauro et al., 2016; Kim, 2017). The criteria used to assess the suitability of a strain are generally the kinetics and total production of acids, pH variation, loss of substances with high nutritional value, decrease in the concentration of nitrates and production of biogenic amines (Karovičová and Kohajdová, 2005). Moreover, the ability of the microbial culture to grow on the substrate, the type of metabolism and its ability to impart desirable sensory properties represent as well selection criteria (Holzapfel, 2002). In recent years, interesting results have been obtained by the use of starter cultures of bacteriocins producing lactic acid bacteria that permitted to obtain more safe, controlled and reproducible vegetable fermentations (Omar et al., 2006). In particular, nisin producer *Lactococcus lactis* strains have been proposed as promising biopreservatives in various foods such as dairy products, meat and ready to eat vegetables and fruits (Siroli et al., 2016; Ho et al., 2018). Nisin was the first bacteriocin characterized and allowed as a food preservative in the European Union (Jones et al., 2005; Siroli et al., 2016). In fact, nisin, and in particular the natural variant Z, has a high solubility and stability in different food systems and a wide antimicrobial spectrum, being particularly effective against Gram-positive bacteria, including *L. monocytogenes, Clostridium* spp. and *Staphylococcus aureus* in different food matrices (Settanni and Corsetti, 2008; Yang et al., 2012; Zhao et al., 2013; Gharsallaoui et al., 2016). However, several studies (Mutaku et al., 2005; de Oliveira Junior et al., 2015) have demonstrated the effects of the addition of nisin in fruit or vegetable juices, but there are few studies about *in situ* production of nisin by strains of *L. lactis*.

In this context, the objective of this study was to evaluate the potential of 15 nisin producers *L. lactis* strains, isolated from milk and dairy products, to be used as fermenting agents of soymilk and carrot juice. For this purpose, the strains were inoculated in the food matrices considered at a level of about 6.0 log CFU/mL and subsequently the growth, acidification and nisin production were evaluated at 20°C. On the three more interesting strains, the effect on the volatile molecule profiles of fermented soymilk and carrot juice was evaluated. Furthermore, the selected strains were screened for the antagonistic activity against a food pathogen frequently associated with vegetable beverages product such as *L. monocytogenes* both in soymilk and carrot juice.

## Materials and Methods

### Microbial Strains and Growth Conditions

Fifteen nisin producing *L. lactis* strains isolated from different niches were used in this study (Table 1). Molecular identification and genotypic characterization were previously performed (Pisano et al., 2015). Part of the strains belong to the collection of Department of Agricultural and Food Sciences, University of Bologna and the others belong to the collection of the Department of Medical Sciences and Public Health, University of Cagliari. As control strain, *L. lactis* subsp. *lactis* ATCC11454 was used (American Type Culture Collection). All the strains were preliminarily grown in M17 broth (Oxoid, Milan, Italy) for 24 h at 30°C, then refreshed two times in M17 broth for 24 h at 20°C before the fermentation trials. The nisin sensitive strain *L. plantarum* V7B3 (Siroli et al., 2016) was used as target microorganism in nisin detection assay. The evaluation of anti-*Listeria* activity was performed against the strain *L. monocytogenes* Scott A. The two strains were preliminarily grown in de Man, Rogosa, and Sharpe (MRS, Oxoid, Milano, Italy) broth and Brain Heart Infusion (BHI, Oxoid, Milano, Italy) broth, respectively, for 24 h at 37°C.

**Table 1:** List of nisin-producing *Lactococcus lactis* strains screened in this study with isolation source and type of nisin produced.

**Strain**	**Species**	**Isolation source**	**Nisin**
3LC39^b^	*Lactococcus lactis* subsp. *lactis*	Goat milk	A
LSGA1B^a^	*Lactococcus lactis* subsp. *lactis/cremoris*	Cow milk	A
11FS16^b^	*Lactococcus lactis* subsp. *lactis*	Fiore sardo cheese	A
9/20234^b^	*Lactococcus lactis* subsp. *lactis*	Sheep milk	A
LSP2^a^	*Lactococcus lactis* subsp. *lactis*	Cow milk	A
LSG3^a^	*Lactococcus lactis* subsp. *lactis/cremoris*	Cow milk	Z
10/18771^b^	*Lactococcus lactis* subsp. *lactis*	Sheep milk	A
LBG1G^a^	*Lactococcus lactis* subsp. *lactis/cremoris*	Cow milk	A
FBG1P^a^	*Lactococcus lactis* subsp. l*actis/cremoris*	Raviggiolo cheese	A
LBG2^a^	*Lactococcus lactis* subsp. l*actis/cremoris*	Cow milk	Z
16FS16^b^	*Lactococcus lactis* subsp. *Lactis*	Fiore sardo cheese	A
1LC18^b^	*Lactococcus lactis* subsp. *Lactis*	Goat milk	A
6LS5^b^	*Lactococcus lactis* subsp. *Lactis*	Sheep milk	Z
6/23898^b^	*Lactococcus lactis* subsp. *Lactis*	Sheep milk	A
ATCC11454	*Lactococcus lactis* subsp. *Lactis*		A

a *Department of Agricultural and Food Sciences, University of Bologna, Italy*.

b *Department of Medical Sciences and Public Health, University of Cagliari, Italy*.

### Soymilk and Carrot Extract Fermentation

Commercial soymilk was used for the fermentation trials. Forty milliliters of drink were poured into sterile tubes and *L. lactis* strains were inoculated at 6.0 log CFU/mL in triplicate and in three different days. Inoculated samples were incubated at 20°C.

Carrot extract was prepared using fresh carrots. The carrots were steeped in a solution containing 100 ppm of Sodium hypochlorite for 2 min for sanitization (Goodburn and Wallace, 2013). Then, were wiped up, sliced and put in a domestic extractor (Russel Hobbs). The resulting extract was collected in a sterile flask and pasteurized at 72°C for 15 min. Then, the extract was aliquoted into 40 mL sterile tubes and *L. lactis* strains were inoculated at 6.0 log CFU/mL in triplicate and in 3 different days. Inoculated samples were incubated at 20°C. Acidification of the samples was evaluated measuring the pH before inoculum and after 7, 24, 48, 72, and 144 h of incubation at 20°C.

### Nisin Activity Determination

Nisin activity was determined in both the food matrices after 72 h of fermentation. Nisin assay was performed by the agar well diffusion method as described by de Oliveira Junior et al. (2015) and Pongtharangkul and Demirci (2004) with some modifications. Ten milliliters of the fermented sample were gathered and pH of the samples was adjusted to 3 with 4N HCl and then centrifuged at 6,000 g for 20 min. The supernatants were collected and filtered (0.45 μm pore diameter). After that, supernatants were boiled for 10 min and then cooled. The supernatants were stored at −20°C until the analyses. The agar well diffusion assay was performed in MRS soft Agar (0.8% agar) inoculated with the nisin sensitive strain *L. plantarum* V7B3 at 7.0 log CFU/mL. Wells of 5 mm diameter were made into each plate and filled with 50 μL of the supernatant. The inhibition zones were measured after incubation for 24 h at 37°C and compared vs. standard concentrations of nisin to obtain concentrations. Control samples considered were both the vegetable drinks at starting pH as well as at pH 4.0 processed similarly to the fermented samples.

A stock nisin solution was prepared by dissolving commercial nisin 2.5% (Sigma-Aldrich, Milan, Italy) into a sterile diluent solution of 0.02 N HCl. Then concentrations, ranging from 1,000 to 0 UI (1,000, 500, 400, 300, 200, 100, 50, and 0 UI/mL), were prepared in soymilk or in carrot extract. The nisin was then extracted by the food matrix as reported above. The activity was plotted against concentration to construct the standard curve. A line regression equation was determined for each standard curve. The activity of nisin expressed in international units per milliliter was converted to mg/L through the relation: nisin (mg/L) = (z × 0.025), where z = IU/mL and 0.025 is a conversion value related to 2.5% pure nisin.

### Screening on the Three Most Interesting Strains

The *L. lactis* strains 3LC39, FBG1P, LBG2 were selected based on the results of the first part of the research and were further scrutinized. The selected strains were used to ferment soymilk and carrot extract at 20°C, in the same way reported in paragraph 2.2, to validate the data obtained in the preliminary screening. Moreover, soymilk and carrot juice inoculated samples were analyzed for nisin activity, according to the method reported in paragraph 2.3, during the fermentation, after 24, 48, 72, and 144 h of incubation at 20°C.

### Challenge Test in the Presence of *Listeria monocytogenes*

The three selected strains (3LC39, FBG1P, LBG2) were scrutinized for the anti-*L. monocytogenes* activity in the two matrices described before. *L. lactis* strains were inoculated at a level of 6.0 log CFU/mL in the matrix (soymilk or carrot juice), while *L. monocytogenes* Scott A was inoculated at 4.0 log CFU/mL, each trial was performed in triplicate. Control samples consist of soymilk or carrot juice inoculated with *L. monocytogenes* (4.0 log CFU/mL) in the absence of *L. lactis* strains. Samples were investigated after 7, 24, 48, 72, and 144 h of incubation at 20°C for the viability of *L. lactis* strains on M17 Agar plates and *L. monocytogenes* on Listeria Selective Agar Base (Oxoid, Milano, Italy) plates.

### Volatile Molecules Profiles

Volatile compounds analysis of the soymilk and carrot juice samples, fermented by the strains 3LC39, FBG1P, LBG2, collected after 24, 48, 72, and 144 h of fermentation, was performed following the method described by Siroli et al. (2017). The Solid-phase microextraction (SPME) fiber used was CAR/PDMS, 75 μm (SUPELCO, Bellafonte, PA, USA). Five milliliters of the sample were placed in vials and incubated for 10 min at 45°C. Then the fiber was exposed to the vial headspace for 30 min at 45°C. The adsorbed volatiles were desorbed in the gas chromatograph (GC) injector port in splitless mode at 250°C for 10 min. The headspace of the volatile compounds was analyzed using gas Gas-Chromatography (GC) 6890N, Network GC System with mass spectrometry (MS) 5970 MSD (Hewlett–Packard, Geneva, Switzerland). The column used was Chrompack CP-Wax 52 CB (50 m × 320 μm × 1.2 μm). The initial temperature was 40°C for 1 min and then increased by 4.5°C/min up to 65°C. After that, the temperature increased by 10°C/min up to 230°C and remain at this temperature for 17 min. Compounds were identified by comparison based on NIST 11 (National Institute of Standards and Technology) database. Gas-carrier was helium at 1.0 mL/min flow.

### Statistical Analysis

Microbiological data, pH-values and the amount of nisin were statistically analyzed using Statistica software (version 8.0; StatSoft, Tulsa, Oklahoma, USA). Means were compared using ANOVA followed by LSD test at *p* < 0.05 level to detect significant differences among the samples. The volatile molecule profiles were analyzed using ANOVA followed by a principal component analysis (PCA) performed using Statistica software (version 8.0; StatSoft, Tulsa, Oklahoma, USA).

## Results

### Preliminary Screening

In the first step of the study, the capability to grow, acidify and produce nisin, of the 15 nisin producer *L. lactis* strains, was evaluated in two different food matrices such as soymilk and carrot juice characterized by different composition and pH-values. In Table 2, the evolution of lactococci counts (log CFU/mL) and pH-values in carrot juice at different stages of storage (fermentation) at 20°C are reported.

**Table 2:** Evolution of lactococci counts (log CFU/mL) and pH-values in carrot juice at different stages of storage (fermentation) at 20°C.

	**log CFU/ml**				**pH**			
**Strain**	**0 h**	**24 h**	**48 h**	**72 h**	**0 h**	**24 h**	**48 h**	**72 h**
3LC39	5.12 ± 0.09^a^	8.29 ± 0.22^a^^b^^c^	9.44 ± 0.14^d^	9.34 ± 0.22^a^	6.50 ± 0.03^a^	4.37 ± 0.13^a^	4.08 ± 0.09^a^^b^	3.98 ± 0.08^a^
LSGA1B	5.45 ± 0.13^b^	7.88 ± 0.17^a^	9.40 ± 0.23^c^^d^	9.31 ± 0.10^a^	6.50 ± 0.03^a^	6.00 ± 0.08^g^	4.09 ± 0.11^a^^b^	3.99 ± 0.07^a^
11FS16	5.66 ± 0.21^b^	8.03 ± 0.23^a^^b^	9.28 ± 0.22^b^^c^^d^	9.39 ± 0.20^a^	6.50 ± 0.03^a^	6.11 ± 0.08^g^	4.09 ± 0.16^a^^b^	4.01 ± 0.11^a^
9/20234	5.67 ± 0.19^b^	7.99 ± 0.11^a^	8.53 ± 0.33^a^	9.26 ± 0.14^a^	6.50 ± 0.03^a^	6.26 ± 0.03^h^	4.68 ± 0.09^c^	4.30 ± 0.11^b^
LSP2	5.67 ± 0.23^b^	8.17 ± 0.22^a^^b^^c^	9.05 ± 0.18^a^^b^^c^	9.22 ± 0.17^a^	6.50 ± 0.03^a^	5.60 ± 0.09^e^^f^	4.07 ± 0.08^a^^b^	3.99 ± 0.09^a^
LSG3	5.38 ± 0.09^b^	8.26 ± 0.20^a^^b^^c^	9.44 ± 0.22^c^^d^	9.31 ± 0.20^a^	6.50 ± 0.03^a^	5.10 ± 0.15^c^^d^	4.15 ± 0.10^a^^b^	4.04 ± 0.04^a^
ATCC11454	5.52 ± 0.05^b^	8.25 ± 0.09^b^	8.97 ± 0.17^a^^b^	9.27 ± 0.18^a^	6.50 ± 0.03^a^	5.47 ± 0.12^e^	4.08 ± 0.08^a^^b^	4.00 ± 0.11^a^
LBG1G	5.44 ± 0.12^b^	8.29 ± 0.10^b^^c^	8.81 ± 0.25^a^^b^	9.19 ± 0.09^a^	6.50 ± 0.03^a^	5.78 ± 0.11^f^	4.07 ± 0.08^a^^b^	4.00 ± 0.10^a^
FBG1P	5.61 ± 0.08^b^	7.86 ± 0.20^a^	8.89 ± 0.21^a^^b^	9.28 ± 0.10^a^	6.50 ± 0.03^a^	4.64 ± 0.06^b^	4.17 ± 0.18^ab^	4.05 ± 0.06^a^
LBG2	5.40 ± 0.22^a^	8.12 ± 0.14^ab^	9.12 ± 0.14^bc^	9.34 ± 0.18^a^	6.50 ± 0.03^a^	4.43 ± 0.05^a^	4.09 ± 0.05^ab^	3.99 ± 0.07^a^
16FS16	5.37 ± 0.11^b^	8.68 ± 0.23^d^	9.16 ± 0.13^bc^	9.36 ± 0.14^a^	6.50 ± 0.03^a^	4.75 ± 0.09^b^	4.24 ± 0.11^b^	4.15 ± 0.12^a^
1LC18	5.59 ± 0.05^b^	8.51 ± 0.15^c^^d^	9.10 ± 0.21^b^^c^^d^	9.22 ± 0.13^a^	6.50 ± 0.03^a^	5.23 ± 0.08^d^	4.06 ± 0.09^a^^b^	3.99 ± 0.14^a^
6LS5	5.42 ± 0.13^b^	8.13 ± 0.21^a^^b^	9.46 ± 0.14^d^	9.38 ± 0.20^a^	6.50 ± 0.03^a^	5.61 ± 0.18^ef^	4.08 ± 0.07^a^^b^	4.00 ± 0.09^a^
6/23898	5.28 ± 0.15^ab^	8.08 ± 0.16^a^^b^	9.10 ± 0.14^b^^c^	9.36 ± 0.20^a^	6.50 ± 0.03^a^	5.00 ± 0.11^c^	3.99 ± 0.10^a^	3.92 ± 0.16^a^
10/18771	5.43 ± 0.07^b^	8.65 ± 0.08^d^	9.14 ± 0.11^b^^c^	9.44 ± 0.22^a^	6.50 ± 0.03^a^	4.59 ± 0.13^a^^b^	3.99 ± 0.08^a^	3.94 ± 0.09^a^

*Means in the same column followed by different letters are significantly different (p < 0.05)*.

The data obtained showed a good fermentation capability of carrot juice (initial pH 6.5) by all the tested lactococci. In fact, they were able to acidify the product nearly the pH-value of 4.0, except for the strain 9/20234, within 48 h of storage at 20°C. However, the strains that showed the significantly (*p* < 0.05) highest acidification rates were 3LC39, LBG2, 10/18771 and, in a minor extent, 16FS16 and FBG1P which were the only ones to show pH-values below 5.0 after 24 h of storage at 20°C. The cell loads of the tested lactococci in carrot juice increased over time. Following the initial increase of 2.5–3.5 log units at 24 h, the lactococci counts reached the maximum at 48 h (about 9.0 log CFU/mL) and then remained stable at 72 h of storage without significant differences among the strains.

In the following step, the same strains were evaluated as potential fermentation starter on a matrix with different nutritional characteristics and pH (6.7) such as soymilk. In this case, the lactococci were inoculated at levels ranging between 5.0 and 6.0 log CFU/mL and the samples were then stored at 20°C. In Table 3 is reported the evolution of lactococci counts (log CFU/mL) and pH-values in soymilk at different stages of storage (fermentation) at 20°C. All the tested strains showed good fermentative characteristics being able to lower the pH mean values of soy beverage by 1.85–2.40 units after 48 h of fermentation at 20°C. The highest acidification rate was observed for the strain LBG2 that showed significantly (*p* < 0.05) lower pH respect to the other strains after 24 h of storage. However, after 48 h the samples inoculated with the strains 3LC39, LSG3, FBG1P, LBG2, 16FS16, and 6LS5 attained the lowest pH-values (*p* < 0.05).

**Table 3:** Evolution of lactococci counts (log CFU/mL) and pH-values in soymilk at different stages of storage (fermentation) at 20°C.

	**log CFU/ml**				**pH**			
**Strain**	**0 h**	**24 h**	**48 h**	**72 h**	**0 h**	**24 h**	**48 h**	**72 h**
3LC39	5.12 ± 0.09^a^	8.49 ± 0.13^bc^	9.22 ± 0.18^a^	9.31 ± 0.11^a^	6.68 ± 0.05^a^	5.35 ± 0.03^c^	4.32 ± 0.09^a^	4.15 ± 0.05^a^
LSGA1B	5.45 ± 0.13^b^	8.58 ± 0.22^bc^	9.11 ± 0.26^a^	9.26 ± 0.24^a^	6.68 ± 0.05^a^	5.77 ± 0.09^e^	4.70 ± 0.08^cd^	4.37 ± 0.08^c^
11FS16	5.66 ± 0.21^b^	8.17 ± 0.18^ab^	9.07 ± 0.20^a^	9.18 ± 0.21^a^	6.68 ± 0.05^a^	6.10 ± 0.07^f^	4.57 ± 0.11^bc^	4.25 ± 0.12^abc^
9/20234	5.67 ± 0.19^b^	8.07 ± 0.23^a^	9.08 ± 0.19^a^	9.22 ± 0.16^a^	6.68 ± 0.05^a^	6.18 ± 0.11^f^	4.83 ± 0.07^d^	4.37 ± 0.09^c^
LSP2	5.67 ± 0.23^b^	8.57 ± 0.15^bc^	9.18 ± 0.09^a^	9.33 ± 0.18^a^	6.68 ± 0.05^a^	5.70 ± 0.12^e^	4.78 ± 0.10^cd^	4.38 ± 0.12^c^
LSG3	5.38 ± 0.09^b^	8.82 ± 0.09^c^	9.26 ± 0.18^a^	9.29 ± 0.09^a^	6.68 ± 0.05^a^	5.30 ± 0.14^bc^	4.44 ± 0.06^ab^	4.13 ± 0.08^a^
ATCC11454	5.52 ± 0.05^b^	8.77 ± 0.17^c^	9.31 ± 0.13^a^	9.34 ± 0.22^a^	6.68 ± 0.05^a^	5.71 ± 0.08^e^	4.77 ± 0.03^d^	4.44 ± 0.13^c^
LBG1G	5.44 ± 0.12^b^	8.61 ± 0.22^bc^	9.21 ± 0.21^a^	9.33 ± 0.33^a^	6.68 ± 0.05^a^	5.75 ± 0.07^e^	4.78 ± 0.07^d^	4.38 ± 0.11^c^
FBG1P	5.61 ± 0.08^b^	8.20 ± 0.19^ab^	9.01 ± 0.36^a^	9.41 ± 0.26^a^	6.68 ± 0.05^a^	5.37 ± 0.09^c^	4.45 ± 0.14^ab^	4.25 ± 0.06^abc^
LBG2	5.40 ± 0.22^a^	8.63 ± 0.23^bc^	9.17 ± 0.22^a^	9.27 ± 0.14^a^	6.68 ± 0.05^a^	4.89 ± 0.11^a^	4.27 ± 0.12^a^	4.13 ± 0.11^ab^
16FS16	5.37 ± 0.11^b^	8.96 ± 0.28^c^	9.36 ± 0.18^a^	9.46 ± 0.19^a^	6.68 ± 0.05^a^	5.15 ± 0.11^b^	4.28 ± 0.12^a^	4.10 ± 0.07^a^
1LC18	5.59 ± 0.05^b^	8.79 ± 0.24^c^	9.24 ± 0.33^a^	9.33 ± 0.08^a^	6.68 ± 0.05^a^	5.65 ± 0.13^de^	4.78 ± 0.07^d^	4.39 ± 0.17^bc^
6LS5	5.42 ± 0.13^b^	8.75 ± 0.09^c^	9.15 ± 0.24^a^	9.28 ± 0.11^a^	6.68 ± 0.05^a^	5.09 ± 0.14^ab^	4.27 ± 0.08^a^	4.11 ± 0.13^ab^
6/23898	5.28 ± 0.15^ab^	8.32 ± 0.22^ab^	9.19 ± 0.27^a^	9.29 ± 0.22^a^	6.68 ± 0.05^a^	5.47 ± 0.09^cd^	4.61 ± 0.09^bc^	4.23 ± 0.11^abc^
10/18771	5.43 ± 0.07^b^	8.86 ± 0.19^c^	9.27 ± 0.22^a^	9.38 ± 0.17^a^	6.68 ± 0.05^a^	5.40 ± 0.04^c^	4.84 ± 0.13^d^	4.45 ± 0.13^c^

*At each time, means followed by different letters are significantly different (p < 0.05)*.

In soymilk all the *L. lactis* strains showed an increase of the cell loads ranging between 3.0 and 4.0 logarithmic cycles after 24 h of storage at 20°C. Within 48 h, all the strains reached the stationary growth phase with cell loads ranging between 9.01 and 9.36 log CFU/mL and without significant differences among the samples.

**Figure 1:**
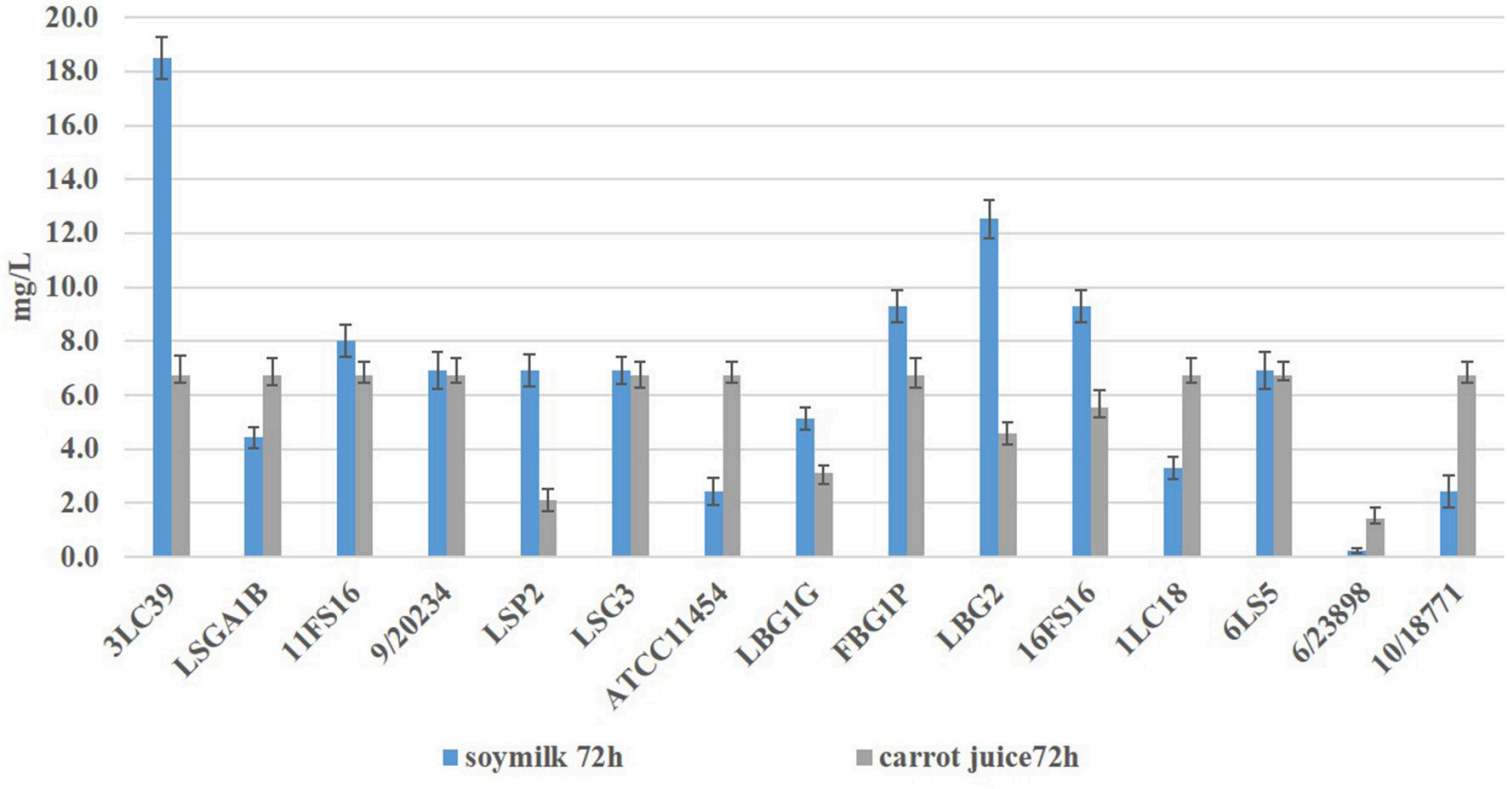
Nisin concentrations (expressed in mg/L ± standard deviation) detected in soymilk and carrot juice after 72 h at 20°C of fermentation by produced the tested *L. lactis* strains.

During the fermentation at 20°C, both in carrot juice and in soymilk, the presence of nisin was detected through an agar well diffusion assay. In Figure 1, the concentrations of nisin (mg/L) detected in soymilk and carrot juice fermented by the tested lactococci after 72 h are reported.

All strains were able to produce nisin in both food matrices in different amount, with the exception of *L. lactis* 6/23898.

In the samples fermented by the strains 3LC39, FBG1P, LBG1G, LBG2, 16FS16, and LSP2, higher amounts of nisin in soymilk than carrot juice after 72 h of storage were detected. The strains LSGA1B, 1LC18, 10/18772 and the type strain ATCC11454 showed an opposite trend. The highest amount of nisin (18.5 ± 0.8 mg/L) was detected in soymilk samples fermented by the strain 3LC39 followed by the strain LBG2 (12.5 ± 0.7 mg/L) and the strains 16FS16 and FBG1P (9.3 ± 0.6 mg/L). By contrast, in carrot juice, except for the samples inoculated with the strains LSP2, LBG1G, LBG2, and 6/23898 the quantities of nisin detected ranged between 5.6 and 6.8 mg/L after 72 h of storage at 20°C.

### Nisin Production, Antimicrobial Activity, and Volatile Molecule Profiles of the Three Selected Lactococci

On the basis of the results of the first experimental part, the strains 3LC39 (highest amount of nisin produced in soymilk and rapid acidification of both the vegetable matrices), LBG2 (fastest acidification of both the vegetable matrices) and FBG1P (rapid acidification and nisin production on both the food matrices) were selected as potential starter cultures for the production of vegetable fermented beverage. In particular, the volatile molecule profiles of the fermented products and the strain antimicrobial activity against *L. monocytogenes* were evaluated both in soymilk and carrot juice.

The fermentation kinetics and the cell loads overtime of the three considered *L. lactis* strains resulted similar to the previous trial (data not shown). The acidification resulted faster in carrot juice than soymilk except for the strain LBG2 that showed similar kinetics on both the matrices.

Table 4 shows the amount of nisin produced by the three lactococci in soymilk and carrot juice at 20°C after 24, 48, 72, and 144 h of storage and expressed as international units of enzymatic activity (IU/mL) as well as mg/L. The amounts of nisin detected in carrot juice were similar after 72 h to the levels detected in preliminary trials for all the strains while in soymilk samples fermented by 3LC39 and FBG1P nisin levels resulted lower respect to those found in the preliminary trials.

**Table 4:** Nisin detected in soymilk and carrot juice in relation to the strain used.

**Strain**	**Matrix**	**24 h**		**48 h**		**72 h**		**144 h**	
		**IU/mL**	**mg/L**	**IU/mL**	**mg/L**	**IU/mL**	**mg/L**	**IU/mL**	**mg/L**
3LC39	Soymilk	532 ± 91	13.3 ± 2.3^b^	532 ± 79	13.3 ± 2.0^b^	532 ± 86	13.3 ± 2.2^a^	268 ± 28	6.7 ± 0.7^b^
FBG1P	Soymilk	323 ± 24	8.1 ± 0.6^d^	268 ± 50	6.7 ± 1.2^d^	268 ± 28	6.7 ± 0.7^b^	268 ± 52	6.7 ± 1.3^b^
LBG2	Soymilk	1055 ± 142	26.4 ± 3.6^a^	1055 ± 188	26.4 ± 4.5^a^	582 ± 73	14.5 ± 1.8^a^	532 ± 63	13.3 ± 1.6^a^
3LC39	Carrot juice	396 ± 10	9.9 ± 0.3^c^	396 ± 20	9.9 ± 0.5^c^	246 ± 63	6.2 ± 1.6^b^	205 ± 37	5.1 ± 0.9^b^
FBG1P	Carrot juice	366 ± 63	9.1 ± 1.6^cd^	396 ± 33	9.9 ± 0.8^c^	246 ± 52	6.2 ± 1.3^b^	141 ± 15	3.5 ± 0.4^c^
LBG2	Carrot juice	396 ± 33	9.9 ± 0.8^c^	246 ± 86	6.2 ± 2.2^d^	205 ± 52	5.1 ± 1.3^b^	141 ± 15	3.5 ± 0.4^c^

*The amount of nisin is expressed as international units (IU/mL) ± standard deviation and converted to mg/L ± standard deviation. At each time, means followed by different letters are significantly different (p < 0.05)*.

In contrast to what observed in preliminary trials, the highest amount of nisin was detected in soymilk samples fermented by the strain LBG2. The mean amount of nisin produced by the LBG2 strain in soymilk at 24, 48 and 144h was significantly (*p* < 0.05) higher than that produced by the other two selected nisin-producer strains. The concentrations of nisin detected in samples fermented by LBG2 decreased after 72 h of storage, when they were almost halved compared to the levels observed after 24 and 48 h. In contrast, in soymilk fermented by the strain 3LC39, nisin was almost constant up to 72 h of storage and decreased after 144 h. Regarding the strain FBG1P, the amounts of nisin detected in fermented soymilk ranged between 8.1 and 6.7 mg/L throughout the whole storage period.

After 24 h, the amounts of nisin detected in carrot samples were similar and independent from the strain used (9.9, 9.9, and 9.1 mg/L for the strains 3LC39, LBG2 and FBG1P, respectively). The nisin amounts in samples fermented by the strains 3LC39 and LBG2 were significantly (*p* < 0.05) lower in carrot juice compared to soymilk. By contrary, in samples fermented by the strain FBG1P, similar amounts of nisin, higher after 48 h of storage, were detected in carrot juice compared to soymilk. Also in carrot juice, the amount of nisin decreased over time. In particular, in carrot juice the decrease of nisin concentration started after 48 h when fermented by LBG2, and after 72 h when fermented by 3LC39 and FBG1P.

**Figure 2:**
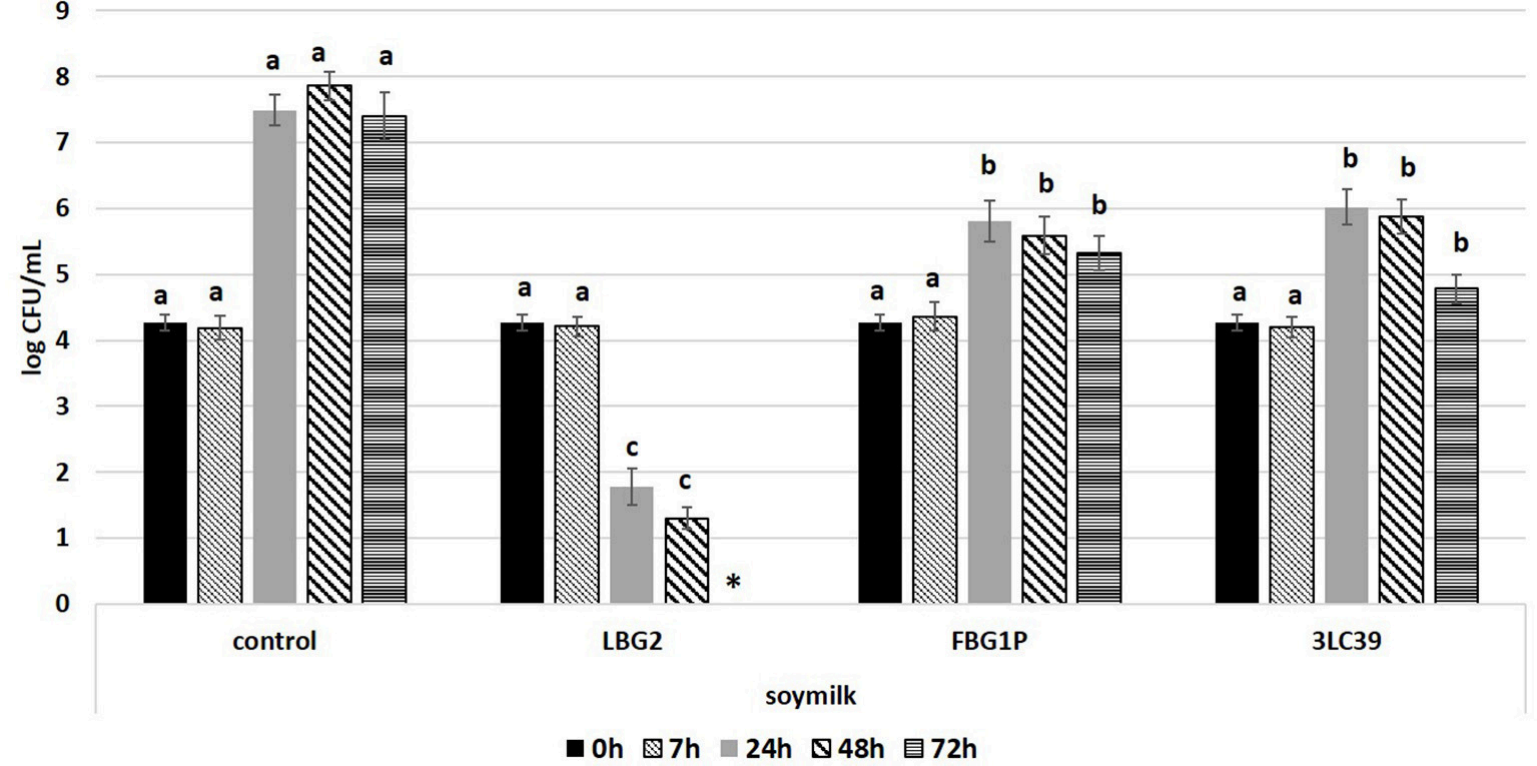
Cell loads (log CFU/mL) of *L. monocytogenes* during the storage at 20°C in soymilk in the presence or absence (control) of the nisin producers *Lactococcus lactis* strains (inoculated at a level of about 5.5 log CFU/mL). The inoculation level of the pathogen was about 4.2 log CFU/mL. Values followed by different letters, at the same time of storage, are significantly different *p* < 0.05. ^*^Under the detection limit.

**Figure 3:**
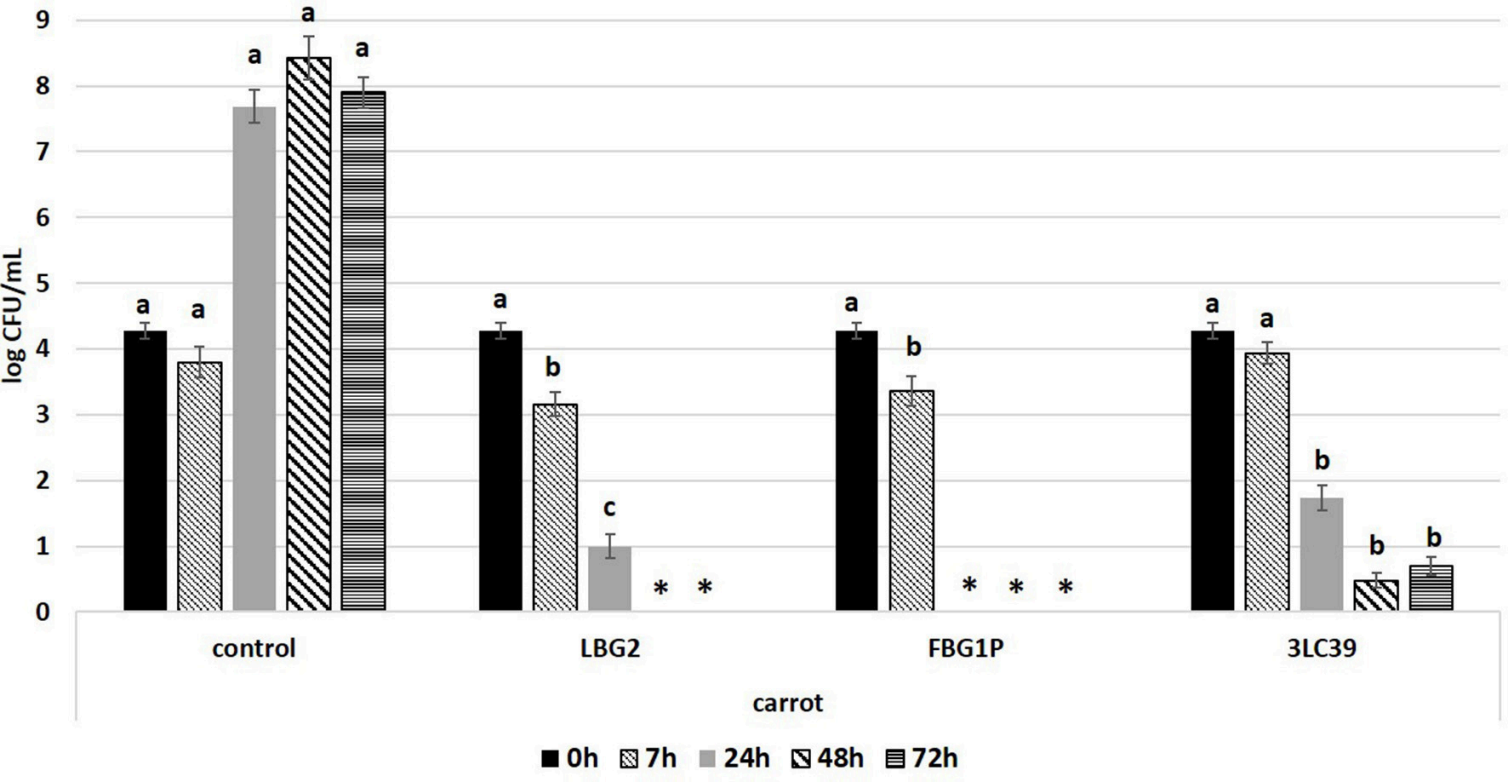
Cell loads (log CFU/mL) of *L. monocytogenes* during the storage at 20°C in carrot juice in the presence or absence (control) of the nisin producers *Lactococcus lactis* strains (inoculated at a level of about 5.5 log CFU/mL). The inoculation level of the pathogen was about 4.2 log CFU/mL. Values followed by different letters, at the same time of storage, are significantly different *p* < 0.05. ^*^Under the detection limit.

Concerning the anti-*Listeria* activity, the lactococci strains considered showed an inhibition of the pathogen depending on the strain and the food matrix. The lactococci, inoculated at a level of 5.5 log CFU/mL, were able to grow independently on the presence of *L. monocytogenes* on both food matrices. In fact, the cell loads of lactococci were similar to those observed in the same matrices in the absence of *Listeria* (data not shown). In Figures 2, 3, the growth of *L. monocytogenes* in soymilk and carrot juice in co-colture with the selected *L. lactis* nisin-producing strains is reported. The control samples were carrot juice or soymilk samples inoculated exclusively with *L. monocytogenes*. In the controls, the *L. monocytogenes* cell loads, starting from 4.3 log CFU/mL, reached a maximum in 48 h then slightly decreased both in soymilk and carrot juice. In soymilk, significantly lower *L. monocytogenes* counts were detected after 48 and 72 h in samples fermented by the three selected lactococci strains and LBG2 showed the best anti-listeria activity. In fact, this strain was able to significantly reduce the *L. monocytogenes* load by 2.5 log units with respect to the initial inoculum and by 5.5 log units with respect to the control at 24 h of incubation. A further significant reduction was observed during incubation until the *L. monocytogenes* load resulted below the detection limit at 72 h.

In fermented carrot juice samples, a higher and faster inhibition of *L. monocytogenes* was observed compared to soymilk (Figure 3). After 7 h, samples fermented by the strains LBG2 and FBG1P showed a significant (*p* < 0.05) decreases in the cell load of the pathogen compared to the other samples. In the controls, *L. monocytogenes* reached the stationary growth phase within 24 h at 20°C. The highest anti-*Listeria* activity was observed in carrot juice fermented by the strain FBG1P. In fact, *Listeria* cell load resulted below the detection limit (0.5 log CFU/mL) already after 24 h indicating decreases higher than 4.0 logarithmic cycles. Regarding the other strains, after 24 h, pathogen cell load decreases higher than 3.0 and 2.5 logarithmic cycles were observed in the samples inoculated with LBG2 and 3LC39, respectively. The pathogen was below the detection limit within 48 h in the samples fermented by the strains LBG2. Although 3LC39 was not able to totally inhibit *Listeria*, the cell loads of the pathogen detected after 48 and 72 h were lower than 1.0 log CFU/ml in the samples co-inoculated with this bio-control agent.

Fermented soymilk and carrot juices were also analyzed for their volatile molecules profiles by GC/MS SPME after 24, 48, 72, and 144 h of storage at 20°C. Tables 5, 6 show the amounts, expressed as equivalent ppm, of all the volatile molecules identified in fermented soymilk and carrot juice, respectively. Fifty-three and sixty-seven different molecules were identified in soymilk and carrot juice samples, respectively. They belonged to several chemical classes such as hydrocarbons, aldehydes, ketones, alcohols, acids, esters, and terpenic compounds. The carrot juice control samples before fermentation had a total concentration of volatile molecules of about 581 equivalent ppm. By contrast, soymilk before fermentation had a total concentration of molecules of about 17 equivalent ppm. In particular, the most representative molecules of carrot juice were terpenic compounds such as β-phellandrene, D-limonene, γ-terpinene, o-cymene, terpinolene, α-caryophyllene, β-trans-Ocimene, elemicin, and myristicin.

**Table 5:** Volatile compounds (expressed as equivalent ppm) detected through GC-MS/SPME technique in soymilk in relation to the strain used and the fermentation time considered at 20°C.

		**3LC39**				**FBG1P**				**LBG2**			
	**T0**	**T24**	**T48**	**T72**	**T144**	**T24**	**T48**	**T72**	**T144**	**T24**	**T48**	**T72**	**T144**
**Molecule**	**ppm**	**ppm**	**ppm**	**ppm**	**ppm**	**ppm**	**ppm**	**ppm**	**ppm**	**ppm**	**ppm**	**ppm**	**ppm**
Acetaldehyde	0.00	0.09	0.10	0.14	0.06	0.08	0.09	0.10	0.06	0.06	0.06	0.07	0.02
2-methyl-butanal	0.02	0.02	0.13	0.12	0.15	0.02	0.08	0.13	0.21	0.21	0.42	0.34	0.31
3-methyl-butanal	0.00	0.00	0.00	0.00	0.00	0.00	0.00	0.00	0.07	1.00	3.10	2.89	2.86
Hexanal	2.32	0.50	1.02	0.88	0.96	0.49	1.16	1.23	1.78	0.91	0.91	0.85	1.31
Heptanal	0.23	0.16	0.23	0.05	0.33	0.10	0.71	0.71	0.50	0.58	0.36	0.44	0.38
Octanal	0.30	0.36	0.56	0.40	0.50	0.27	0.57	0.83	0.84	1.21	1.25	1.12	1.02
Nonanal	0.26	0.47	0.63	0.46	0.52	0.33	0.66	0.67	0.95	1.61	1.12	1.31	0.99
Decanal	0.09	0.20	0.34	0.16	0.17	0.18	0.16	0.25	0.27	0.47	0.37	0.34	0.22
Furfural	0.00	0.00	0.00	0.18	0.11	0.00	0.11	0.22	0.32	0.00	0.00	0.10	0.36
Benzaldehyde	0.07	0.35	0.51	0.23	0.49	0.29	0.24	0.42	0.67	0.32	0.24	0.38	1.11
Benzeneacetaldehyde	0.00	0.00	0.00	0.00	0.00	0.00	0.00	0.00	0.00	0.20	0.28	0.29	0.50
**Total aldehydes**	**3.30**	**2.16**	**3.53**	**2.62**	**3.28**	**1.76**	**3.79**	**4.57**	**5.66**	**6.56**	**8.11**	**8.13**	**9.10**
Furan 2-Ethyl	0.15	0.04	0.09	0.04	0.12	0.03	0.09	0.09	0.15	0.08	0.06	0.07	0.10
2-Butylfuran	0.00	0.00	0.00	0.00	0.00	0.00	0.04	0.04	0.05	0.04	0.13	0.03	0.03
2-pentyl-furan	0.34	0.22	0.38	0.39	0.45	0.18	0.47	0.47	0.67	0.39	0.36	0.28	0.58
2,4,4,6,6,8,8-heptamethyl-1-nonene	0.25	0.33	0.44	0.07	0.44	0.26	0.46	0.51	0.69	0.51	0.45	0.43	0.40
2,4,4,6,6,8,8-heptamethyl-2-nonene	0.27	0.34	0.66	0.57	0.47	0.21	0.39	0.44	0.70	0.43	0.54	0.60	0.21
**Total hydrocarbons**	**1.01**	**0.92**	**1.57**	**1.07**	**1.48**	**0.69**	**1.45**	**1.56**	**2.25**	**1.46**	**1.54**	**1.40**	**1.33**
2-Butanone	0.00	0.00	0.12	0.16	0.36	0.00	0.31	0.35	0.33	0.00	0.00	0.07	0.20
2-Pentanone	0.21	0.06	0.11	0.13	0.16	0.05	0.16	0.28	0.38	0.07	0.14	0.14	0.19
Diacetyl	0.00	0.39	0.99	1.70	2.38	0.23	1.36	1.70	4.55	0.29	0.88	0.99	1.78
methyl isobutyl ketone	0.42	0.57	0.67	0.55	0.54	0.75	0.70	0.49	0.37	0.56	0.73	0.62	0.42
4-methyl-2-hexanone	0.29	0.42	0.46	0.32	0.34	0.37	0.48	0.42	0.41	0.46	0.26	0.43	0.34
4-methyl-3-penten-2-one	0.41	0.71	0.78	0.78	0.40	0.99	1.01	0.83	0.76	1.22	0.75	0.82	0.55
2,6-dimethyl-4-heptanone	0.11	0.39	0.33	0.32	0.29	0.28	0.33	0.24	0.26	0.40	0.27	0.22	0.22
2-heptanone	0.00	0.06	0.11	0.15	0.20	0.09	0.13	0.12	0.22	0.12	0.13	0.15	0.23
2-heptanone, 4-methyl	0.00	0.03	0.06	0.06	0.15	0.00	0.07	0.09	0.18	0.00	0.00	0.00	0.00
3-Octanone	0.00	0.00	0.06	0.09	0.13	0.00	0.13	0.14	0.20	0.00	0.00	0.00	0.09
Acetoin	0.00	0.53	0.94	1.84	2.04	0.12	0.73	2.09	3.51	1.18	2.29	3.33	3.10
2-Nonanone	0.00	0.00	0.09	0.14	0.54	0.00	0.00	0.00	0.00	0.14	0.11	0.10	0.08
**Total ketons**	**1.44**	**3.15**	**4.71**	**6.22**	**7.52**	**2.87**	**5.40**	**6.75**	**11.15**	**4.44**	**5.57**	**6.87**	**7.19**
Ethanol	1.05	1.34	1.82	1.99	2.41	1.25	1.68	1.84	2.39	1.43	1.51	1.76	2.01
2-hexanol 2,3 dimethyl	0.31	0.20	0.69	0.56	0.84	0.13	0.74	0.86	1.08	0.76	0.66	0.70	0.60
3-methyl-1-butanol	0.00	0.00	0.00	0.00	0.00	0.03	0.15	0.22	0.52	0.79	2.98	2.63	4.79
2-hexanol	0.18	0.27	0.28	0.21	0.11	0.29	0.30	0.24	0.19	0.28	0.15	0.14	0.18
5-methyl-3-hexanol	0.13	0.27	0.18	0.18	0.16	0.21	0.26	0.18	0.13	0.22	0.17	0.17	0.19
1-pentanol	0.12	0.07	0.19	0.17	0.24	0.12	0.21	0.24	0.46	0.22	0.25	0.15	0.27
2,4,4-trimethyl-1-pentanol	0.12	0.27	0.60	0.00	0.00	0.23	0.36	0.35	0.56	0.40	0.37	0.80	0.27
1-hexanol	0.19	0.58	1.19	2.05	2.32	0.56	1.31	1.90	4.33	0.77	0.94	1.22	2.01
3-octanol	0.00	0.00	0.00	0.11	0.14	0.00	0.00	0.00	0.00	0.23	0.19	0.18	0.00
1-octen-3-ol	0.57	0.59	0.59	1.35	1.54	0.52	0.98	1.05	1.70	0.64	0.83	0.90	1.50
Heptanol	0.00	0.00	0.00	0.09	0.10	0.00	0.09	0.10	0.15	0.06	0.08	0.09	0.10
1-hexanol, 2-Ethyl	0.00	0.00	0.00	0.12	0.16	0.00	0.10	0.12	0.16	0.00	0.00	0.08	0.16
3-Nonanol	0.00	0.25	0.39	0.25	0.20	0.19	0.21	0.25	0.33	0.49	0.38	0.42	0.14
1-Octanol	0.00	0.12	0.26	0.08	0.15	0.08	0.16	0.21	0.28	0.26	0.21	0.20	0.13
1,2-Decandiol	0.00	0.00	0.00	0.00	0.00	0.00	0.00	0.00	0.00	0.29	0.21	0.24	0.05
3,6,9,12-tetraoxatetradecan-1-ol	0.04	0.25	0.29	0.00	0.28	0.12	0.18	0.22	0.24	0.33	0.27	0.24	0.15
2-methyl-3-octanol	0.00	0.08	0.15	0.13	0.11	0.00	0.10	0.34	0.25	0.26	0.29	0.20	0.21
Benzyl alcohol	0.00	0.00	0.00	0.00	0.00	0.00	0.00	0.00	0.29	0.00	0.00	0.14	0.15
phenethyl alcohol	0.00	0.00	0.00	0.00	0.00	0.00	0.00	0.00	0.37	0.26	0.84	0.81	0.51
**Total alcohols**	**2.72**	**4.29**	**6.63**	**7.30**	**8.77**	**3.74**	**6.84**	**8.12**	**13.42**	**7.70**	**10.34**	**11.05**	**13.42**
Ethyl acetate	0.00	0.03	0.03	0.03	0.04	0.00	0.11	0.05	0.04	0.00	0.02	0.02	0.04
cyclohexilmethyl tetradecyl ester	1.21	1.12	1.30	1.73	1.82	1.18	1.26	1.86	2.44	1.88	1.17	1.31	1.41
**Totale esters**	**1.21**	**1.15**	**1.33**	**1.76**	**1.86**	**1.18**	**1.37**	**1.90**	**2.48**	**1.88**	**1.18**	**1.33**	**1.45**
Acetic acid	0.00	0.52	1.77	2.09	2.50	0.52	0.61	2.02	3.20	2.28	5.20	4.95	3.19
Pentanoic acid 4-methyl-2-oxo	0.00	0.00	0.00	0.00	0.00	0.00	0.16	0.04	0.00	0.00	1.22	0.89	1.05
Pentanoic acid, 2-hydroxy 4-methyl	0.00	0.00	0.00	0.00	0.00	0.00	0.00	0.00	0.00	2.70	2.33	2.26	3.51
Hexanoic acid	0.00	0.00	0.45	0.34	0.27	0.10	0.21	0.30	0.83	0.53	0.70	0.61	0.41
**Total acids**	**0.00**	**0.52**	**2.22**	**2.43**	**2.77**	**0.62**	**0.98**	**2.36**	**4.03**	**5.51**	**9.45**	**8.70**	**8.16**
**Total molecules**	**17.22**	**15.26**	**31.39**	**18.74**	**30.86**	**13.37**	**25.93**	**30.94**	**48.78**	**38.62**	**43.41**	**44.38**	**42.91**

**Table 6:** Volatile compounds (expressed as ppm) detected through GC-MS/SPME technique in carrot juice in relation to the strain used and the fermentation time considered at 20°C.

		***L. lactis*** **FBG1P**				***L. lactis*** **3LC39**				***L. lactis*** **LBG2**			
	**T0**	**T24**	**T48**	**T72**	**T144**	**T24**	**T48**	**T72**	**T144**	**T24**	**T48**	**T72**	**T144**
**Molecule**	**ppm**	**ppm**	**ppm**	**ppm**	**ppm**	**ppm**	**ppm**	**ppm**	**ppm**	**ppm**	**ppm**	**ppm**	**ppm**
2-pentyl-furan	0.36	0.18	0.42	0.45	0.57	0.27	0.39	0.47	0.30	0.23	0.50	0.36	0.40
3,6-dimethyl-benzofuran	0.00	0.42	0.82	1.70	1.94	0.25	1.12	1.62	1.68	0.14	1.03	1.21	2.44
α-pinene	6.45	2.83	2.66	2.42	1.94	5.86	3.20	2.64	1.52	5.48	2.63	1.91	1.20
camphene	0.31	0.00	0.00	0.00	0.00	0.57	0.00	0.34	0.22	0.18	0.28	0.28	0.10
β-pinene	4.63	2.18	2.05	1.89	1.83	4.91	2.82	1.89	1.23	3.48	2.59	1.40	1.28
β-phellandrene	9.37	3.34	2.72	2.29	1.28	4.59	2.63	2.02	0.86	5.07	2.78	1.56	1.28
α-phellandrene	4.15	3.03	1.98	1.73	1.48	4.04	3.29	2.43	1.83	3.55	2.88	2.22	1.95
α-terpinene	2.40	1.80	1.49	1.12	1.05	2.14	1.42	1.26	0.87	1.89	1.58	1.11	1.01
D-limonene	19.17	12.88	9.77	8.65	7.04	17.41	9.36	8.66	5.76	12.26	9.92	9.88	8.22
Ocimene	1.10	0.29	0.23	0.47	0.34	0.58	0.46	0.41	0.25	0.47	0.40	0.17	0.53
γ-terpinene	46.71	31.07	27.30	24.56	20.09	41.73	29.10	26.11	17.63	36.41	33.24	28.54	23.17
o-cymene	13.70	11.62	10.58	10.23	9.54	12.54	10.91	8.91	7.67	10.53	9.69	9.16	8.61
terpinolene	85.73	78.94	75.98	65.95	57.06	82.07	75.69	70.23	53.55	77.89	71.98	62.12	60.77
*p*-menthatriene	1.97	1.11	0.93	0.98	0.60	1.49	1.22	0.97	0.33	0.90	0.89	0.55	0.53
*p*,α-Dimethylstyrene	5.45	5.17	5.04	4.42	3.51	5.28	4.95	4.98	2.85	4.45	3.93	3.56	3.77
caryophyllene	87.99	79.47	71.55	68.93	62.54	85.46	72.96	67.54	64.25	82.92	76.13	68.54	66.21
β-bisabolene	3.34	3.03	2.32	2.02	1.53	3.10	2.50	2.64	1.73	3.25	2.86	1.79	1.55
α-caryophyllene	11.98	8.88	7.86	6.87	6.92	10.94	7.15	6.22	6.15	9.67	8.08	6.73	6.88
α-bisabolene	2.00	1.09	0.95	1.33	1.85	1.83	1.04	1.79	1.03	1.31	1.15	1.01	1.01
β-trans-Ocimene	22.31	22.28	20.49	18.40	16.88	23.21	20.45	17.45	14.56	18.94	17.39	15.54	14.02
α-curcumene	0.89	0.65	0.54	0.55	0.52	0.98	0.43	0.69	0.59	0.79	0.53	0.58	0.80
Caryophyllene oxide	0.00	0.00	0.64	1.90	3.03	0.54	1.64	1.99	2.57	0.66	1.59	1.89	2.47
Elemicin	13.52	12.31	12.52	12.17	11.98	8.77	8.32	7.90	7.58	8.85	8.56	8.13	8.22
Myristicin	203.8	198.2	177.5	158.5	144.7	197.1	193.3	181.9	157.5	191.3	185.3	175.8	167.4
**Total hydrocarbons**	**547.9**	**481.2**	**437.4**	**398.7**	**359.3**	**515.9**	**455.3**	**422.1**	**353.2**	**480.8**	**446.7**	**404.8**	**384.3**
Acetaldehyde	0.00	0.14	0.12	0.13	0.12	0.12	0.15	0.16	0.15	0.12	0.13	0.13	0.14
2-methyl-butanal	0.03	0.06	0.13	0.14	0.16	0.07	0.09	0.11	0.13	0.09	0.11	0.14	0.17
3-methyl-butanal	0.00	0.76	1.02	1.40	1.46	0.03	0.04	0.04	0.04	0.73	0.77	1.00	1.14
hexanal	0.71	0.33	0.56	0.59	1.18	0.52	0.69	1.07	1.15	0.35	0.54	0.98	1.34
heptanal	0.36	0.45	0.77	1.39	1.19	0.73	1.60	1.77	1.16	0.20	1.20	1.11	1.22
nonanal	2.21	2.98	2.28	2.19	1.38	2.81	2.24	2.02	1.40	2.17	1.89	1.82	1.66
decanal	0.00	0.55	0.51	0.62	0.87	0.11	0.42	0.61	1.02	0.30	0.67	0.57	0.64
3-nonanal	0.00	0.69	0.83	0.85	0.81	0.37	0.94	1.11	1.17	0.59	0.72	0.86	0.89
2-nonenal, (*e*)	1.32	1.31	1.49	1.72	1.73	1.56	1.61	1.37	1.03	0.78	0.98	1.44	1.71
Benzenacetaldehyde	0.00	0.44	0.65	0.98	0.94	0.43	0.37	0.32	0.34	0.32	0.38	0.24	0.67
**Total aldehydes**	**4.63**	**7.70**	**8.36**	**10.00**	**9.84**	**6.75**	**8.14**	**8.58**	**7.59**	**5.66**	**7.38**	**8.29**	**9.58**
2-Butanone	0.17	0.29	0.35	0.38	0.36	0.27	0.33	0.35	0.35	0.17	0.29	0.36	0.37
Dyacetil	0.00	0.45	0.55	1.12	1.64	0.42	0.59	1.10	2.38	0.30	0.75	1.09	4.31
methyl isobutyl ketone	0.23	0.30	0.34	0.29	0.27	0.25	0.23	0.31	0.36	0.34	0.30	0.33	0.31
4-methyl-3-penten-2-one	0.41	0.48	0.54	0.51	0.47	0.58	0.86	0.95	0.81	0.33	0.51	0.88	0.61
2,6-dimethyl-4-heptanone	0.00	0.00	0.45	0.57	0.96	0.00	0.59	0.71	0.68	0.24	0.75	0.79	0.89
2,2 dimethyl-3-Octanone	0.46	0.10	0.13	0.25	0.27	0.00	0.11	0.17	0.13	0.00	0.30	0.14	0.24
acetoin	0.00	0.00	0.00	0.00	0.00	0.47	1.14	1.54	2.18	0.00	0.00	0.68	0.85
6-methyl-5-hepten-2-one	0.00	0.00	0.00	0.49	0.95	0.00	0.48	0.56	0.66	0.00	0.44	0.34	0.69
2-nonanone	0.00	0.39	0.53	0.66	0.86	0.52	0.58	0.61	0.64	0.23	0.46	0.37	0.72
Geranyl acetone	0.00	0.00	0.00	0.00	1.01	0.00	0.55	0.71	0.81	0.00	0.78	0.64	0.78
**Total ketons**	**1.27**	**2.00**	**2.90**	**4.28**	**6.79**	**2.51**	**5.46**	**7.02**	**9.01**	**1.61**	**4.56**	**5.60**	**9.78**
Ethanol	0.86	2.12	1.89	1.85	1.71	2.27	1.95	1.71	1.78	1.87	1.83	1.98	2.09
Cyclopentanol	0.06	0.07	0.07	0.00	0.00	0.08	0.12	0.22	0.21	0.06	0.08	0.19	0.39
3-methyl-1-butanol	0.00	0.39	1.04	1.33	1.41	0.00	0.14	0.18	0.23	0.83	0.98	1.00	1.47
1-heptanol	0.00	0.34	0.45	0.68	1.13	0.36	0.77	0.77	0.86	0.25	0.43	0.46	0.72
2-ethylhexanol	0.00	0.16	0.17	0.17	0.32	0.12	0.23	0.46	0.44	0.12	0.18	0.18	0.38
2,6-dimethyl-4-heptanol	0.00	1.25	1.09	1.19	1.28	0.12	0.00	0.00	0.68	0.91	1.06	0.72	0.86
1-octanol	0.00	1.73	1.94	2.30	3.91	1.70	2.75	2.70	2.84	1.12	1.96	2.09	3.14
terpinen-4-ol	0.18	1.96	3.89	5.44	6.89	2.13	4.48	5.99	6.09	0.90	4.32	5.50	7.21
2-Cyclohexen-1-ol	0.18	0.29	0.87	0.95	1.30	0.18	0.83	1.00	1.45	0.14	0.53	0.57	0.57
*p*-Cimen-8-ol	0.00	1.02	1.16	1.66	3.02	1.71	1.58	1.81	1.72	0.59	1.35	1.43	1.78
Eugenol	5.01	4.64	4.53	4.12	3.65	4.51	3.92	3.15	2.33	3.73	2.58	2.64	2.67
2-Methoxy-4-vinyl-phenol	0.00	0.80	0.68	0.53	0.09	0.00	0.30	0.00	0.00	0.41	0.36	0.32	0.20
α-methyl-benzenemethanol	0.75	1.23	1.65	1.75	1.40	0.51	1.49	1.32	0.93	0.34	1.06	1.08	0.50
methyl eugenol	1.15	1.40	1.39	1.47	1.35	1.41	1.50	1.29	0.90	1.65	1.36	1.10	0.96
**Total alcohols**	**8.19**	**17.42**	**20.82**	**23.43**	**27.46**	**15.11**	**20.05**	**20.59**	**20.46**	**12.90**	**18.06**	**19.27**	**22.95**
acetic acids	0.00	1.14	1.63	1.83	2.12	1.45	1.91	2.25	2.22	0.78	1.59	1.90	1.56
tetradecyl oxalic acid	2.44	2.31	3.36	3.36	3.96	1.79	3.39	2.92	2.14	1.15	2.57	2.43	1.73
ester thiophene-2-acetic acid	5.45	5.82	7.19	8.97	9.10	5.78	8.62	9.06	7.96	5.07	8.08	8.73	7.75
octanoic acid	0.00	0.00	0.00	0.00	0.66	0.00	0.00	0.00	0.30	0.23	0.63	0.40	0.39
**Total acids**	**7.88**	**9.27**	**12.17**	**14.17**	**15.83**	**9.02**	**13.92**	**14.22**	**12.63**	**7.23**	**12.87**	**13.45**	**11.44**
Thymol methyl ester	4.56	2.57	2.60	2.57	2.52	3.98	2.96	2.67	1.65	2.66	2.65	1.98	2.21
L-α-bornyl acetate	4.84	4.15	3.82	4.24	3.21	5.37	5.31	5.17	2.47	3.96	3.99	2.80	2.99
Linalyl 3-methylbutanoate	0.00	0.00	0.00	0.00	0.00	1.71	0.58	1.12	0.51	0.26	0.59	0.50	0.63
**Totale ester**	**9.40**	**6.72**	**6.41**	**6.81**	**5.73**	**11.06**	**8.85**	**8.96**	**4.63**	**6.89**	**7.23**	**5.28**	**5.83**
**Total molecules**	**579.3**	**524.3**	**488.0**	**457.3**	**425.0**	**560.3**	**511.7**	**481.4**	**407.5**	**515.1**	**496.8**	**456.7**	**443.9**

Contrarily, not inoculated soymilk presented very low quantities of volatile molecules and they were mainly aldehydes. As evidenced in Tables 5, 6, the fermentation processes led to specific volatile profiles in relation to the food matrix, the strain and the time of storage considered. As expected, in soymilk a qualitative-quantitative increase in volatile molecules was observed during the fermentation process. In particular, in all the fermented samples, significant increases of acids (i.e., acetic acid), alcohols (mainly ethanol, 3-methyl 1-butanol, and 1-hexanol), ketones (mainly diacetyl and acetoin) and aldehydes were found. Although all the lactococci strains increased the complexity of the volatile molecule profiles of fermented soymilk, the GC-MS-SPME profiles were influenced by the strain. The strain LBG2 led to a higher production of aldehydes (3-methyl-butanal, octanal, nonanal), acids (acetic acid and pentanoic acid) and alcohols (3-methyl-1 butanol) compared to the other strains. On the contrary, samples fermented by the strain FBG1P were characterized by greater amounts of ketones, in particular, diacetyl, acetoin and esters. Finally, soymilk samples fermented by 3LC39 showed the lower concentrations of volatile molecules compared to the other samples.

The fermented carrot juices showed different volatile molecule profiles depending on the considered strain. In general, a significant reduction of the initial concentrations of terpenic molecules was observed. On the contrary, a notable increase of aldehydes, ketones, alcohols and acids was detected. The kinetics of quantitative reduction of the terpenic molecules during the storage were similar for all the lactococci considered. However, the increase in the amount of the other classes of molecules was strain dependent. The major qualitative and quantitative differences among the strains concerned the chemical class of ketones. The strain LBG2 was characterized by the highest levels of diacetyl, while the strain 3LC39 gave raise to the highest amounts of acetoin. By contrast, the strain FBG1P was characterized by the lowest concentrations of ketones and by the absence of acetoin.

**Figure 4:**
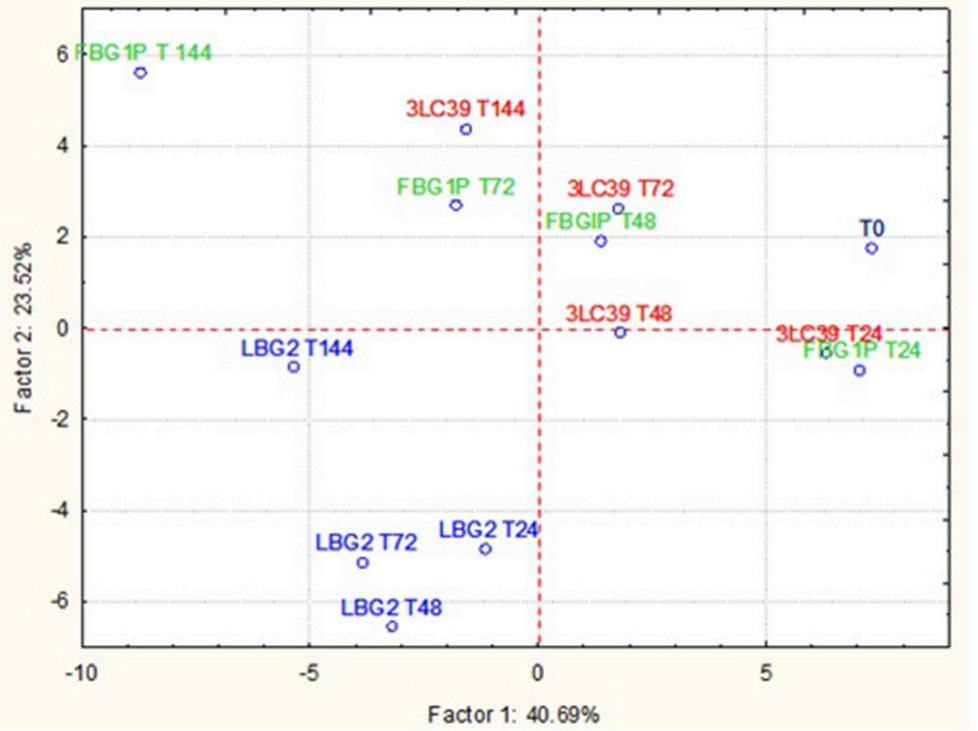
Projection of the cases on the factor plane (1X2), soymilk samples at a different time (24, 48, 72, and 144 h) fermented by three different nisin producers *L. lactis* strains at 20°C. PC1 and PC2 explained 40.69 and 23.52% of the total variance, respectively.

The GC-MS-SPME raw data were subjected to a principal component analysis (PCA) to better highlight the differences in the volatile molecule profiles of fermented soymilk and carrot juice in relation to the *L. lactis* strain used. In Figure 4 is reported the projection of cases on the factorial plane (1x2) spanned by first two factors (PC1 and PC2) of soymilk samples. The projection of the samples on the factorial plane was affected by the *L. lactis* strain used and the time of storage considered. All samples fermented by the strain LBG2 were separated from the others both along the PC1, which explains 40.69% of the total variance, and along the PC2, which explains 23.52% of the variance. Furthermore, samples fermented with this latter strain were very similar to each other after 24, 48, and 72 h. Only the samples after 144 h were well-separated from the others, but mainly along the PC2. Also the samples fermented by 3LC39 and FBG1P strains were similar among them but separated according to the storage time considered. In addition, after 24 h the soymilk samples fermented with the strains 3LC39 and FBG1P resulted very similar to the control samples (T0). The increase of the storage time led to a shift of the samples mainly along the PC1. Regarding the molecules that allowed the clustering of the fermented samples, the soymilk fermented with the strain LBG2 were characterized by aldehydes (3-methyl-butanal, octanal, nonanal), acids (acetic acid and pentanoic acid), and alcohols (3-methyl-1-pentanol). On the contrary, the soymilk fermented with FBG1P and 3LC39 strains were clustered mainly by the ketones (data not shown).

**Figure 5:**
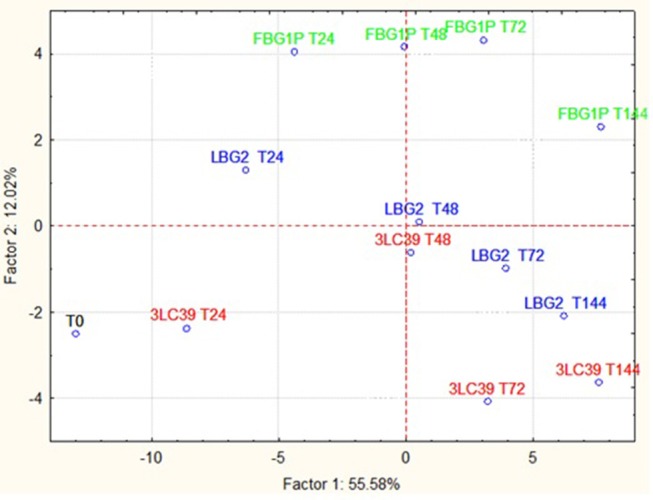
Projection of the cases on the factor plane (1X2), carrot juice samples at a different time (24, 48, 72, and 144 h) fermented by three different nisin producers *L. lactis* strains at 20°C. PC1 and PC2 explained 55.58 and 12.02% of the total variance, respectively.

In Figure 5, the projection of the cases on the factorial plane define by PC1 and PC2 relative to the carrot juice samples is shown. In this case, the samples significantly differed in relation to the *L. lactis* strain used and the storage time considered. However, the samples fermented by the different strains were separated from each other, at the same storage time, mainly along the PC2, which explains only 12.02% of the total variance. The samples at different storage times were separated from each other along the PC1, which explains 55.58% of the total variance. In this case, the clustering of the carrot juices fermented with the strains LBG2 and 3LC39 was mainly due to the presence of ketones such as diacetyl and acetoin. On the contrary, the clustering of fermented samples by FBG1P was due to the highest concentrations of aldehydes and alcohols.

## Discussion

The data obtained highlighted rapid acidification of soymilk by all the tested strains, especially the strain *L. lactis* LBG2. These results confirm that the compositional characteristics of soymilk (high pH-values, macro and micronutrient availability) are optimal for the growth of lactococci (Beasley et al., 2003; Granato et al., 2010). However, the faster acidification induced by the strain LBG2, compared to the other strains, can be attributed to the capability to use nutrients that is a strain dependent characteristic (Linares et al., 2017).

Also in carrot juice, the *L. lactis* strains considered showed good potential as fermenting agents. The cell loads of the strains increased very quickly and as well as the acidification of the vegetable beverage was faster than those observed with the other strains. This feature is very important to demonstrate the potential application of *L. lactis* strains for the fermentation of vegetable drinks. In fact, a fast acidification kinetic is a prerequisite for the strains to be used as a starter and it is essential to prevent the possible contamination of spoiling microorganisms and pathogens (Di Cagno et al., 2013; Filannino et al., 2014). From this point of view, the lowering of the pH, the production of organic acids and bacteriocins represent important hurdles to prevent possible contamination by undesirable microorganisms, including spoilage and pathogenic ones (O'Sullivan et al., 2002). Furthermore, rapid fermentation kinetics are also essential for the potential industrial application of the selected strains. Rapid fermentation production processes significantly reduce the costs both in terms of energy and work (Leroy and De Vuyst, 2004).

The data obtained both in the first and in the second experimental phase, confirm that the production of nisin in food systems is extremely variable depending on the *L. lactis* strain considered and, above all, the composition of the food matrix (Beasley et al., 2003; de Arauz et al., 2009; Liu et al., 2017). The strains LBG2 and 3LC39 produced a higher amount of nisin in soymilk than carrot juice, while the strain FBG1P produced a similar amount of nisin in both the food matrices. In particular, for the strain LBG2, despite similar growth kinetics in carrot juice and soymilk, the nisin activity detected was three times higher in the latter matrix. On the other hand, literature data show that the presence of micronutrients, such as potassium and calcium salts and vitamins, such as B2 and B12, of which soymilk is enriched, are essential factors that can quantitatively influence the capability to produce nisin by lactococci (Li et al., 2002; Beasley et al., 2003). Few data are available about nisin production by *L. lactis* strains in vegetable drinks. However, several studies reported the application of nisin as food additive in different juices such as tomato, kiwi, peach, apple, mango, and orange normally at a concentration ranging between 0.5 and 60 mg/L (de Oliveira Junior et al., 2015). In addition, Jiangbo et al. (2017) showed that the concentration of 20 IU/mL of nisin was enough to inhibit different species of *Alicyclobacillus* in kiwi juice. Pathanibul et al. (2009) showed more than 5.0 log reduction of *Listeria innocua* in carrot juice by combining high pressure homogenization treatment (350 MPa) and nisin used at a concentration of 10 IU/mL. Given the above, the amount of nisin detected in our trials can be considered satisfactory since resulted higher respect to the concentrations of nisin reported in literature as effective in this kind of products.

All samples considered in the present research showed a more or less marked decrease in the amount of nisin detected over time independently on the raw material and strain considered. This trend confirms the literature data indicating that the major part of nisin production occurs in the late exponential growth phase and the beginning of the stationary phase. Subsequently, a reduction of the metabolic process leading to the production of the bacteriocin takes place since its biosynthesis is inhibited by the bacteriocin accumulation in the growth media (Abbasiliasi et al., 2017). Furthermore, the chemical-physical and compositional characteristics of the substrate are notoriously capable of modifying the stability and activity of the bacteriocin over time. The bacteriocin can interact with other macromolecules present in the substrate, losing its antimicrobial activity (Fernández-Pérez et al., 2018; Silva et al., 2018). Furthermore, nisin is susceptible to depletion due to its physical diffusion within the food system or its degradation by proteases (Sarkar et al., 2017). The effect of the food matrix on the stability and activity of nisin is also evident in the data obtained in this experimentation. In fact, nisin resulted more stable in carrot juice than in soymilk, although the production was higher in the latter matrix.

The results obtained on the *anti-Listeria* activity of the three strains of *L. lactis* LBG2, FBG1P and 3LC39 in soymilk are in agreement with the strain growth kinetics and the amount of nisin produced. The strain LBG2, was the only able to totally inhibit *Listeria* in soymilk. On the other hand, it was the one that showed the fastest acidification as well as the greater production of nisin. Although a high production of nisin was also detected for the strains 3LC39 and FBG1P, they were not able to completely inhibit *L. monocytogenes*. Only partial inhibition was observed for these strains. Moreover, compared to the strain LBG2 the strains FBG1P and 3LC39 showed a slower decrease in pH which after 24 h was still above 5.0. These pH-values were not able to completely inhibit *L. monocytogenes*, which, thanks to the composition of soymilk extremely rich in micro and macronutrients fundamental for microbial growth, was able to grow also in the presence of the nisin producing strains 3LC39 and FBG1P. In addition, the strain *L. monocytogenes* Scott A used in this work is reported to be quite resistant to nisin (Schillinger et al., 2001). In particular, Schillinger et al. (2001) showed that the amount of nisin necessary to cause a significant reduction of *L. monocytogenes* Scott A in tofu was 3,000 IU/mL. This amount could be reduced by combining nisin with other mild technology or by reducing the pH-value of the matrix. The totally inhibition of *L. monocytogenes* Scott A in soymilk fermented by the strain LBG2 represents a very interesting result and confirm the higher effectiveness of using a nisin producer strain compared to use nisin alone, due to the production of other antimicrobials such as organic acids.

The anti-*Listeria* activity of the lactococci strains in carrot juice was extremely high for all the strains tested, despite the amounts of nisin produced over time were lower than those detected in soymilk. In particular, the strains LBG1P and LBG2 were able to totally inhibit *Listeria* within 24 and 48 h, respectively. However, the increased antimicrobial activity of the *L. lactis* strains in carrot juice can be attributed to the faster acidification observed in this product. All the samples fermented by the three *L. lactis* strains reached pH-values lower than 4.5 within 24 h of fermentation. The higher acidity achieved in the carrot juice samples compared to soymilk ones is certainly the most limiting factor for the growth of *L. monocytogenes*. Literature data indicate that *L. monocytogenes* growth is strongly limited by low pH-values both in model and in food systems (Razavi Rohani et al., 2011; Dal Bello et al., 2012; Aryani et al., 2015). Moreover, Boziaris and Nychas (2006) showed that the minimum pH at which growth of *L. monocytogenes* Scott A was observed was 4.81 in model system, and the addition of 100 IU/ml of nisin increased the minimum pH for growth at 5.20. Besides, the literature shows that the efficacy and stability of nisin are higher at more acidic conditions and lower pH-values (Gharsallaoui et al., 2016). These factors have certainly contributed to the greater effectiveness of lactococci against *L. monocytogenes* in carrot juice compared to soymilk, despite the amount of nisin produced was higher in the latter food matrix. Also, the nisin concentrations detected over time confirmed the greater stability of this bacteriocin in carrot juice compared to soymilk.

The samples of carrot juice showed a significantly higher amount of volatile molecules compared to soymilk samples. This difference was due to the massive presence in carrot juice of terpene molecules components of carrot essential oil. In fact, over 90% of the molecules detected were represented by these compounds. On the other hand, the carrot juice samples were subjected before fermentation process only to a mild heat treatment allowing to the terpenic molecules to remain in the product while soymilk samples were subjected to a sterilization process that affects the presence of volatile molecules. In both the matrices, an increase in alcohols and ketones amounts was observed at the end of the storage due to the fermentation by the *L. lactis* strains. Alcohols are normally produced as end products in the degradation of glucose and catabolism of amino acids, are reported to positively affect the aroma in vegetable juices and can act as the solvent for other aromatic compounds resulting in a greater contribution to the overall aroma (Chen et al., 2019). Ketones are produced by the microbial oxidation of fatty acids or by decarboxylation pathways and are described by intense aroma, even at small concentrations (Chen et al., 2019). Both in soymilk and carrot juice fermentation with the *L. lactis* strains 3LC39, FBG1P, and LBG2 led to specific profiles in volatile molecules in relation to the food matrix, strain, and storage time. The differences can be attributed to the metabolism and the release of molecules with a positive organoleptic impact by the *L. lactis* strains during fermentation (Kwaw et al., 2018). In soymilk, the fermentation with lactococci allowed the appearance of newly formed molecules such as diacetyl and acetoin which are reported as characteristics of fermented cheeses and milk, where they represent key molecules to impart good sensory profiles to the products (Clark and Winter, 2015; Siroli et al., 2017). The PCA of the fermented soymilk showed a clustering of the samples both based on the *L. lactis* strain and the time of storage. On the other hand, the growth kinetics of a lactic acid bacteria strongly affect the profile of volatile molecules of the fermented product (Ricci et al., 2018). In particular, the strains LBG2 showed a rapid increase of the amount of volatile molecule particularly of aldehydes (3-methyl-butanal) alcohols (3-methyl-1-butanol) and acids (acetic acid). The alcohol 3-methyl-1-butanol has been reported to contribute to a fruity and molasses sensation (Tripathi et al., 2014) while 3-methyl-butanal is associated with ethereal and aldehydic notes (Ricci et al., 2018). Considering that currently, the safety and stabilization of soymilk is obtained with severe heat treatments that determine a deterioration of the organoleptic characteristics of the products that represent the main factor of rejection by the consumer (Potter et al., 2007), the improvement of the sensory profile of a heat-treated product represent an important goal.

The fermentation of carrot juice by the *L. lactis* strains increased the presence of aldehydes, ketones, alcohols and acids reported with a positive impact on the product flavor (Fukuda et al., 2013). In particular, the characteristics neo-formed molecules during the fermentation process were 3-methyl butanal, acetoin, diacetyl, 3-methyl-1-butanol, 1-heptanol, 1-octanol, and acetic acid. These volatile molecules have been previously associated to a positive sensory impact in different fermented juices (Filannino et al., 2014; Di Cagno et al., 2017; Ricci et al., 2018).

Also, in the fermented carrot juices, a significant quantitative reduction of the initially present terpenic molecules was observed, mainly due to microbial detoxification of molecules such as terpinolene, limonene, and myristicin. These data are in agreement with the decrease of terpenic molecules in carrot juice observed by other authors during the fermentation process and the refrigerated storage (Fukuda et al., 2013). In addition, myristicin and elemicin are reported as anti-nutritional compounds potentially cause of toxicological symptoms (Dolan et al., 2010). The significant and fast decrease of these molecules, observed in samples fermented by *L. lactis* strains represents a very important result for the improvement of the nutritional properties of the fermented carrot juice. On the other hand, the use of lactic acid fermentation as food detoxification process is extensively reported for neutralize anti-nutritional factors such as phytates, saponins, tannins, cyanogens, or trypsin inhibitors (Septembre-Malaterre et al., 2018).

## Conclusion

The results obtained highlighted the interesting properties of different strains of *L. lactis* as fermenting agents of soymilk and carrot juice. In particular, the strains LBG2, 3LC39 and FBG1P showed a rapid acidification of the considered vegetable beverages and they were capable of producing nisin in the considered food matrices improving their safety due to its anti-*Listeria* activity. In fact, the strain LBG2 was able to totally inhibit *Listeria* both in soymilk and carrot juice. Furthermore, these results underlined the good potential of the selected *L. lactis* strains to improve the sensory profiles of these fermented beverages, suggesting an improvement of the organoleptic properties of pre-heat treated samples. Therefore, although the results obtained are extremely interesting, further experiments are necessary to establish the consumer's acceptance, the shelf-life and the amount of bioactive compound of these fermented products.

## Author Contributions

LS contributed to the experimental design of the work, the acquisition and analysis of the obtained results and the writing of the manuscript. LC contributed to the acquisition and analysis of the obtained results. MP contributed to the writing and the critical revision of the manuscript. FP and RL contributed to the experimental design of the work, the interpretation of the obtained results and the writing and the critical revision of the manuscript.

### Conflict of Interest Statement

The authors declare that the research was conducted in the absence of any commercial or financial relationships that could be construed as a potential conflict of interest.

## References

[B1] Abbasiliasi S. Tan J. S. Tengku Ibrahim T. A. Bashokouh F. Ramakrishnan N. R. Mustafa S. (2017). Fermentation factors influencing the production of bacteriocins by lactic acid bacteria: a review. RSC Adv. 7, 29395–29420. 10.1039/C6RA24579J

[B2] Aryani D. C. den Besten H. M. W. Hazeleger W. C. Zwietering M. H. (2015). Quantifying strain variability in modeling growth of *Listeria monocytogenes*. Int. J. Food Microbiol. 208, 19–29. 10.1016/j.ijfoodmicro.2015.05.006 26011600

[B3] Beasley S. Tuorila H. Saris P. E. (2003). Fermented soymilk with a monoculture of *Lactococcus lactis*. Int. J. Food Microbiol. 81, 159–162. 10.1016/S0168-1605(02)00196-4 12457590

[B4] Bello E. F. T. Martínez G. G. Ceberio B. F. K. Rodrigo D. López A. M. (2014). High pressure treatment in foods. Foods 3, 476–490. 10.3390/foods3030476 28234332 PMC5302251

[B5] Berger C. N. Sodha S. V. Shaw R. K. Griffin P. M. Pink D. Hand P.. (2010). Fresh fruit and vegetables as vehicles for the transmission of human pathogens. Environ. Microbiol. 12, 2385–2397. 10.1111/j.1462-2920.2010.02297.x 20636374

[B6] Bevilacqua A. Petruzzi L. Perricone M. Speranza B. Campaniello D. Sinigaglia M. (2018). Nonthermal technologies for fruit and vegetable juices and beverages: overview and advances. Compr. Rev. Food Sci. Food Saf. 17, 2–62. 10.1111/1541-4337.12299 33350062

[B7] Boziaris I. S. Nychas G.-J. E. (2006). Effect of nisin on growth boundaries of *Listeria monocytogenes* Scott a, at various temperatures, pH and water activities. Food Microbiol. 23, 779–784. 10.1016/j.fm.2006.03.003 16943082

[B8] Callejón R. M. Rodríguez-Naranjo M. I. Ubeda C. Hornedo-Ortega R. Garcia-Parrilla M. C. Troncoso A. M. (2015). Reported foodborne outbreaks due to fresh produce in the United States and European Union: trends and causes. Foodborne Pathog. Dis. 12, 32–38. 10.1089/fpd.2014.1821 25587926

[B9] Cao Z.-H. Green-Johnson J. M. Buckley N. D. Lin Q.-Y. (2018). Bioactivity of soy-based fermented foods: a review. Biotechnol. Adv. 37, 223–238. 10.1016/j.biotechadv.2018.12.001 30521852

[B10] Chen C. Lu Y. Yu H. Chen Z. Tian H. (2019). Influence of 4 lactic acid bacteria on the flavor profile of fermented apple juice. Food Biosci. 27, 30–36. 10.1016/j.fbio.2018.11.006

[B11] Clark S. Winter C. K. (2015). Diacetyl in foods: a review of safety and sensory characteristics. Compr. Rev. Food Sci. Food Saf. 14, 634–643. 10.1111/1541-4337.12150

[B12] Dal Bello B. Cocolin L. Zeppa G. Field D. Cotter P. D. Hill C. (2012). Technological characterization of bacteriocin producing *Lactococcus lactis* strains employed to control *Listeria monocytogenes* in Cottage cheese. Int. J. Food Microbiol. 153, 58–65. 10.1016/j.ijfoodmicro.2011.10.016 22104121

[B13] de Arauz L. J. Jozala A. F. Mazzola P. G. Vessoni Penna T. C. (2009). Nisin biotechnological production and application: a review. Trends Food Sci. Technol. 20, 146–154. 10.1016/j.tifs.2009.01.056

[B14] de Oliveira Junior A. A. Silva de Araújo Couto H. G. Barbosa A. A. T. Carnelossi M. A. G. de Moura T. R. (2015). Stability, antimicrobial activity, and effect of nisin on the physico-chemical properties of fruit juices. Int. J. Food Microbiol. 211, 38–43. 10.1016/j.ijfoodmicro.2015.06.029 26162590

[B15] Di Cagno R. Coda R. De Angelis M. Gobbetti M. (2013). Exploitation of vegetables and fruits through lactic acid fermentation. Food Microbiol. 33, 1–10. 10.1016/j.fm.2012.09.003 23122495

[B16] Di Cagno R. Filannino P. Gobbetti M. (2017). Lactic acid fermentation drives the optimal volatile flavor-aroma profile of pomegranate juice. Int. J. Food Microbiol. 248, 56–62. 10.1016/j.ijfoodmicro.2017.02.014 28244373

[B17] Dolan L. C. Matulka R. A. Burdock G. A. (2010). Naturally occurring food toxins. Toxins 2, 2289–2332. 10.3390/toxins2092289 22069686 PMC3153292

[B18] Fernández-Pérez R. Sáenz Y. Rojo-Bezares B. Zarazaga M. Rodríguez J. M. Torres C.. (2018). Production and antimicrobial activity of nisin under enological conditions. Front. Microbiol. 9:1918. 10.3389/fmicb.2018.01918 30233504 PMC6134021

[B19] Filannino P. Cardinali G. Rizzello C. G. Buchin S. De Angelis M. Gobbetti M.. (2014). Metabolic responses of *Lactobacillus plantarum* strains during fermentation and storage of vegetable and fruit juices. Appl. Environ. Microbiol. 80, 2206–2215. 10.1128/AEM.03885-13 24487533 PMC3993129

[B20] Fukuda T. Tanaka H. Ihori H. Okazaki K. Shinano T. Fukumori Y. (2013). Note Identification of Important Volatiles in Fresh and Processing Carrot Varieties: Using Kuroda and Flakee Types. Available online at: http://chemdata (Accessed October 30, 2018).

[B21] Gharsallaoui A. Oulahal N. Joly C. Degraeve P. (2016). Nisin as a food preservative: part 1: physicochemical properties, antimicrobial activity, and main uses. Crit. Rev. Food Sci. Nutr. 56, 1262–1274. 10.1080/10408398.2013.763765 25675115

[B22] Goodburn C. Wallace C. A. (2013). The microbiological efficacy of decontamination methodologies for fresh produce: a review. Food Control 32, 418–427. 10.1016/j.foodcont.2012.12.012

[B23] Granato D. Branco G. F. Cruz A. G. Faria J. de A. F. Shah N. P. (2010). Probiotic dairy products as functional foods. Compr. Rev. Food Sci. Food Saf. 9, 455–470. 10.1111/j.1541-4337.2010.00120.x 33467833

[B24] Ho V. T. T. Lo R. Bansal N. Turner M. S. (2018). Characterisation of *Lactococcus lactis* isolates from herbs, fruits and vegetables for use as biopreservatives against *Listeria monocytogenes* in cheese. Food Control 85, 472–483. 10.1016/j.foodcont.2017.09.036

[B25] Holzapfel W. H. (2002). Appropriate starter culture technologies for small-scale fermentation in developing countries. Int. J. Food Microbiol. 75, 197–212. 10.1016/S0168-1605(01)00707-3 12036143

[B26] Jiangbo Z. Xinghua L. Tianli Y. Yahong Y. (2017). The effect of nisin on the growth and heat resistance of *Alicyclobacillus acidoterrestris*, *Alicyclobacillus herbarius* and *Alicyclobacillus contaminans* in Kiwi Juice. J. Food Process. Preserv. 41:e13051 10.1111/jfpp.13051

[B27] Jones E. Salin V. Williams G. W. (2005). Nisin and the Market For Commericial Bacteriocins. Available online at: https://ageconsearch.umn.edu/bitstream/90779/2/CP 01 05 Nisin Report.pdf (Accessed October 29, 2018).

[B28] Kaneko S. Kumazawa K. Nishimura O. (2011). Studies on the key aroma compounds in soy milk made from three different soybean cultivars. J. Agric. Food Chem. 59, 12204–12209. 10.1021/jf202942h 21981068

[B29] Karovičová J. Kohajdová Z. (2005). Lactic acid fermentation of various vegetable juices. Acta Aliment. 34, 237–246. 10.1556/AAlim.34.2005.3.5

[B30] Kim S. Y. (2017). Production of fermented kale juices with lactobacillus strains and nutritional composition. Prev. Nutr. Food Sci. 22, 231–236. 10.3746/pnf.2017.22.3.231 29043222 PMC5642806

[B31] Kregiel D. (2015). Health safety of soft drinks: contents, containers, and microorganisms. Biomed. Res. Int. 2015, 1–15. 10.1155/2015/128697 25695045 PMC4324883

[B32] Kubo M. T. K. Augusto P. E. D. Cristianini M. (2013). Effect of high pressure homogenization (HPH) on the physical stability of tomato juice. Food Res. Int. 51, 170–179. 10.1016/j.foodres.2012.12.004

[B33] Kumar S. Thippareddi H. Subbiah J. Zivanovic S. Davidson P. M. Harte F. (2009). Inactivation of *Escherichia coli* K-12 in apple juice using combination of high-pressure homogenization and chitosan. J. Food Sci. 74, M8–M14. 10.1111/j.1750-3841.2008.00974.x 19200108

[B34] Kwaw E. Ma Y. Tchabo C. Apaliya M. T. Wu M. Sackey A. S.. (2018). Effect of lactobacillus strains on phenolic profile, color attributes and antioxidant activities of lactic-acid-fermented mulberry juice. Food Chem. 250, 148–154. 10.1016/j.foodchem.2018.01.009 29412905

[B35] Leroy F. De Vuyst L. (2004). Lactic acid bacteria as functional starter cultures for the food fermentation industry. Trends Food Sci. Technol. 15, 67–78. 10.1016/j.tifs.2003.09.004

[B36] Li C. Bai J. Cai Z. Ouyang F. (2002). Optimization of a cultural medium for bacteriocin production by *Lactococcus lactis* using response surface methodology. J. Biotechnol. 93, 27–34. 10.1016/S0168-1656(01)00377-7 11690692

[B37] Linares D. M. Gómez C. Renes E. Fresno J. M. Tornadijo M. E. Ross R. P.. (2017). Lactic acid bacteria and bifidobacteria with potential to design natural biofunctional health-promoting dairy foods. Front. Microbiol. 8:846. 10.3389/fmicb.2017.00846 28572792 PMC5435742

[B38] Liu J. Zhou J. Wang L. Ma Z. Zhao G. Ge Z.. (2017). Improving nitrogen source utilization from defatted soybean meal for nisin production by enhancing proteolytic function of *Lactococcus lactis* F44. Sci. Rep. 7:6189. 10.1038/s41598-017-06537-w 28733629 PMC5522456

[B39] Mauro C. Guergoletto K. Garcia S. Mauro C. S. I. Guergoletto K. B. Garcia S. (2016). Development of blueberry and carrot juice blend fermented by *Lactobacillus reuteri* LR92. Beverages 2:37 10.3390/beverages2040037

[B40] Medic J. Atkinson C. Hurburgh C. R. (2014). Current knowledge in soybean composition. J. Am. Oil Chem. Soc. 91, 363–384. 10.1007/s11746-013-2407-9

[B41] Mutaku I. Erku W. Ashenafi M. (2005). Growth and survival of *Escherichia coli* O157:H7 in fresh tropical fruit juices at ambient and cold temperatures. Int. J. Food Sci. Nutr. 56, 133–139. 10.1080/09637480500082439 16019323

[B42] Nadeem M. Ubaid N. Qureshi T. M. Munir M. Mehmood A. (2018). Effect of ultrasound and chemical treatment on total phenol, flavonoids and antioxidant properties on carrot-grape juice blend during storage. Ultrason. Sonochem. 45, 1–6. 10.1016/j.ultsonch.2018.02.034 29705302

[B43] Omar N. Ben Abriouel H. Lucas R. Martínez-Cañamero M. Guyot J.-P. Gálvez A. (2006). Isolation of bacteriocinogenic *Lactobacillus plantarum* strains from ben saalga, a traditional fermented gruel from Burkina Faso. Int. J. Food Microbiol. 112, 44–50. 10.1016/j.ijfoodmicro.2006.06.014 16844251

[B44] O'Sullivan L. Ross R. Hill C. (2002). Potential of bacteriocin-producing lactic acid bacteria for improvements in food safety and quality. Biochimie 84, 593–604. 10.1016/S0300-9084(02)01457-8 12423803

[B45] Pathanibul P. Taylor T. M. Davidson P. M. Harte F. (2009). Inactivation of *Escherichia coli* and *Listeria innocua* in apple and carrot juices using high pressure homogenization and nisin. Int. J. Food Microbiol. 129, 316–320. 10.1016/j.ijfoodmicro.2008.12.020 19167772

[B46] Patrignani F. Tabanelli G. Siroli L. Gardini F. Lanciotti R. (2013). Combined effects of high pressure homogenization treatment and citral on microbiological quality of apricot juice. Int. J. Food Microbiol. 160, 273–281. 10.1016/j.ijfoodmicro.2012.10.021 23290235

[B47] Patrignani F. Vannini L. Kamdem S. L. S. Lanciotti R. Guerzoni M. E. (2009). Effect of high pressure homogenization on *Saccharomyces cerevisiae* inactivation and physico-chemical features in apricot and carrot juices. Int. J. Food Microbiol. 136, 26–31. 10.1016/j.ijfoodmicro.2009.09.021 19828206

[B48] Pferschy-Wenzig E.-M. Getzinger V. Kunert O. Woelkart K. Zahrl J. Bauer R. (2009). Determination of falcarinol in carrot (*Daucus carota* L.) genotypes using liquid chromatography/mass spectrometry. Food Chem. 114, 1083–1090. 10.1016/j.foodchem.2008.10.042

[B49] Pisano M. B. Fadda M. E. Melis R. Ciusa M. L. Viale S. Deplano M. (2015). Molecular identification of bacteriocins produced by *Lactococcus lactis* dairy strains and their technological and genotypic characterization. Food Control 51, 1–8. 10.1016/j.foodcont.2014.11.005

[B50] Pongtharangkul T. Demirci A. (2004). Evaluation of agar diffusion bioassay for nisin quantification. Appl. Microbiol. Biotechnol. 65, 268–272. 10.1007/s00253-004-1579-5 14963617

[B51] Potter R. M. Dougherty M. P. Halteman W. A. Camire M. E. (2007). Characteristics of wild blueberry–soy beverages. LWT Food Sci. Technol. 40, 807–814. 10.1016/j.lwt.2006.04.006

[B52] Razavi Rohani S. M. Moradi M. Mehdizadeh T. Saei-Dehkordi S. S. Griffiths M. W. (2011). The effect of nisin and garlic (*Allium sativum* L.) essential oil separately and in combination on the growth of *Listeria monocytogenes*. LWT Food Sci. Technol. 44, 2260–2265. 10.1016/j.lwt.2011.07.020

[B53] Rekha C. R. Vijayalakshmi G. (2010). Bioconversion of isoflavone glycosides to aglycones, mineral bioavailability and vitamin B complex in fermented soymilk by probiotic bacteria and yeast. J. Appl. Microbiol. 109, 1198–1208. 10.1111/j.1365-2672.2010.04745.x 20477889

[B54] Ricci A. Cirlini M. Levante A. Dall'Asta C. Galaverna G. Lazzi C. (2018). Volatile profile of elderberry juice: effect of lactic acid fermentation using *L. plantarum, L. rhamnosus* and *L. casei* strains. Food Res. Int. 105, 412–422. 10.1016/j.foodres.2017.11.042 29433231

[B55] Riciputi Y. Serrazanetti D. I. Verardo V. Vannini L. Caboni M. F. Lanciotti R. (2016). Effect of fermentation on the content of bioactive compounds in tofu-type products. J. Funct. Foods 27, 131–139. 10.1016/j.jff.2016.08.041

[B56] Sarkar P. Bhunia A. K. Yao Y. (2017). Impact of starch-based emulsions on the antibacterial efficacies of nisin and thymol in cantaloupe juice. Food Chem. 217, 155–162. 10.1016/j.foodchem.2016.08.071 27664621

[B57] Schillinger U. Becker B. Vignolo G. Holzapfel W. H. (2001). Efficacy of nisin in combination with protective cultures against *Listeria monocytogenes* scott A in tofu. Int. J. Food Microbiol. 71, 159–168. 10.1016/S0168-1605(01)00612-2 11789934

[B58] Septembre-Malaterre A. Remize F. Poucheret P. (2018). Fruits and vegetables, as a source of nutritional compounds and phytochemicals: changes in bioactive compounds during lactic fermentation. Food Res. Int. 104, 86–99. 10.1016/j.foodres.2017.09.031 29433787

[B59] Serrazanetti D. I. Ndagijimana M. Miserocchi C. Perillo L. Guerzoni M. E. (2013). Fermented tofu: enhancement of keeping quality and sensorial properties. Food Control 34, 336–346. 10.1016/j.foodcont.2013.04.047

[B60] Settanni L. Corsetti A. (2008). Application of bacteriocins in vegetable food biopreservation. Int. J. Food Microbiol. 121, 123–138. 10.1016/j.ijfoodmicro.2007.09.001 18022269

[B61] Sharma K. D. Karki S. Thakur N. S. Attri S. (2012). Chemical composition, functional properties and processing of carrot—a review. J. Food Sci. Technol. 49, 22–32. 10.1007/s13197-011-0310-7 23572822 PMC3550877

[B62] Silva C. C. G. Silva S. P. M. Ribeiro S. C. (2018). Application of bacteriocins and protective cultures in dairy food preservation. Front. Microbiol. 9:594. 10.3389/fmicb.2018.00594 29686652 PMC5900009

[B63] Siroli L. Patrignani F. Serrazanetti D. I. Parolin C. Palomino R. A. N. Vitali B.. (2017). Determination of antibacterial and technological properties of vaginal lactobacilli for their potential application in dairy products. Front. Microbiol. 8:166. 10.3389/fmicb.2017.00166 28223974 PMC5293754

[B64] Siroli L. Patrignani F. Serrazanetti D. I. Vannini L. Salvetti E. Torriani S. (2016). Use of a nisin-producing *Lactococcus lactis* strain, combined with natural antimicrobials, to improve the safety and shelf-life of minimally processed sliced apples. Food Microbiol. 54, 11–19. 10.1016/j.fm.2015.11.004 25583340

[B65] Tamminen M. Salminen S. Ouwehand A. C. (2013). Fermentation of Carrot Juice by Probiotics: Viability and Preservation of Adhesion. Available online at: http://www.lifescienceglobal.com/pms/index.php/ijbwi/article/viewFile/637/541 (Accessed October 29, 2018).

[B66] Terhaag M. M. Almeida M. B. Benassi M. de T (2013). Soymilk plain beverages: correlation between acceptability and physical and chemical characteristics. Food Sci. Technol. 33, 387–394. 10.1590/S0101-20612013005000052

[B67] Timmermans R. A. H. Nederhoff A. L. Nierop Groot M. N. van Boekel M. A. J. S. Mastwijk H. C. (2016). Effect of electrical field strength applied by PEF processing and storage temperature on the outgrowth of yeasts and moulds naturally present in a fresh fruit smoothie. Int. J. Food Microbiol. 230, 21–30. 10.1016/j.ijfoodmicro.2016.04.014 27116618

[B68] Tremarin A. Brandão T. R. S. Silva C. L. M. (2017). Inactivation kinetics of *Alicyclobacillus acidoterrestris* in apple juice submitted to ultraviolet radiation. Food Control 73, 18–23. 10.1016/j.foodcont.2016.07.008

[B69] Tripathi J. Chatterjee S. Gamre S. Chattopadhyay S. Variyar P. S. Sharma A. (2014). Analysis of free and bound aroma compounds of pomegranate (*Punica granatum* L.). LWT Food Sci. Technol. 59, 461–466. 10.1016/j.lwt.2014.05.055

[B70] Yang E. Fan L. Jiang Y. Doucette C. Fillmore S. (2012). Antimicrobial activity of bacteriocin-producing lactic acid bacteria isolated from cheeses and yogurts. AMB Express 2:48. 10.1186/2191-0855-2-48 22963659 PMC3488010

[B71] Zhang Y. Liu X. Wang Y. Zhao F. Sun Z. Liao X. (2016). Quality comparison of carrot juices processed by high-pressure processing and high-temperature short-time processing. Innov. Food Sci. Emerg. Technol. 33, 135–144. 10.1016/j.ifset.2015.10.012

[B72] Zhao L. Wang S. Liu F. Dong P. Huang W. Xiong L. (2013). Comparing the effects of high hydrostatic pressure and thermal pasteurization combined with nisin on the quality of cucumber juice drinks. Innov. Food Sci. Emerg. Technol. 17, 27–36. 10.1016/j.ifset.2012.10.004

